# Word choice is coupled with grammar: The case of kinship

**DOI:** 10.1371/journal.pone.0355784

**Published:** 2026-08-14

**Authors:** Danielle Barth, Nicholas Evans, Andrea C. Schalley, Lila San Roque, Sonja Gipper, John Mansfield, Gabrielle Hodge, Alan Rumsey, I. Wayan Arka, Henrik Bergqvist, Gregory Dickson, Christian Döhler, Diana Forker, Volker Gast, Dolgor Guntsetseg, Eri Kashima, Yukinori Kimoto, Dominique Knuchel, Norikazu Kogura, Keita Kurabe, Heiko Narrog, Desak Putu Eka Pratiwi, Stefan Schnell, Chikako Senge, Elena Skribnik, Saskia van Putten

**Affiliations:** 1 School of Culture, History & Language, Australian National University, Canberra, Australian Capital Territory, Australia; 2 Centre of Excellence for the Dynamics of Language, Australian National University, Canberra, Australian Capital Territory, Australia; 3 Department of Language, Literature and Intercultural Studies, Karlstad University, Karlstad, Sweden; 4 Department of Linguistics, University of Cologne, Cologne, Germany; 5 Institute for the Interdisciplinary Study of Language Evolution, University of Zurich, Zurich, Switzerland; 6 Department of Linguistics and English Language, University of Edinburgh, Scotland, United Kingdom; 7 Department of Linguistics, Stockholm University, Stockholm, Sweden; 8 Berlin-Brandenburg Academy of Sciences and Humanities, Berlin, Germany; 9 Institute for Slavonic Languages and Caucasus Studies, Friedrich Schiller University of Jena, Jena, Germany; 10 Department of English and American Studies, Friedrich Schiller University Jena, Jena, Germany; 11 Institute of Finno-Ugric and Uralic Studies, Ludwig Maximilian University Munich, Munich, Germany; 12 Graduate School of Humanities, Osaka University, Japan; 13 Department of Linguistics, University of Bern, Bern, Switzerland; 14 Research Institute for Languages and Cultures of Asia and Africa, Tokyo University of Foreign Studies, Fuchu-shi, Tokyo, Japan; 15 Faculty of Arts and Letters, Tohoku University, Aoba-ku, Sendai, Japan; 16 Faculty of Foreign Languages, Universitas Mahasaraswati Denpasar, Denpasar, Bali, Indonesia; 17 Centre for East Asian Studies, Faculty of Social Sciences, University of Turku, Turku, Finland; 18 Centre for Language Studies, Radboud University Nijmegen, Netherlands; University of Passau: Universitat Passau, GERMANY

## Abstract

Is it true that ‘grammars code best what speakers do most’, as Du Bois suggested already in 1985? And do members of different cultures privilege talk about kinship relations to differing degrees? Could this be linked to the differential centrality of kinship to the vastly diverse grammars of the world’s languages? We bring these three questions together in this study. Kinship relationships are central to the organisation of all human societies. Yet, we know little about what influences the frequency of reference to kin in spontaneous language use. In this study, we test factors that potentially associate with frequency of kin-term usage across 22 typologically diverse languages. Using a communicative task involving the description, arrangement and narration of pictures, we establish the proportion of human reference expressions formulated as kin terms in interactive language use and show that it does indeed differ across languages. We then examine candidate predictors for the usage frequency of kin-based lexical formulations (choice of word categories) by looking at (i) a structural variable (‘kintax’, the presence of grammatical means for encoding kinship in the language), (ii) a lexical variable (complexity of a subset of each language’s kinship term system), (iii) a discourse variable (type of interaction a language user engages in at the time of speaking or signing), and (iv) a topicality variable (type of stimulus a language user references at the time of speaking or signing). We find all these to be relevant, but we focus on structural kintax. This provides a way of addressing Du Bois’s hypothesis about the interaction between language structure and language use in human communication: the grammatical imprint of a language correlates with language users’ word-choice patterns. We show that the more grammar you have for this semantic domain, the more you talk about it.

## Introduction

One of the most important things people do with language is talk about other people. Through reference to ourselves and to other humans we share social information and encode, enact, and alter social relations. Even though human reference is a universal behavior, it is also a system with many choices [1]. The choices that a person has in any given moment in talk (how will I refer to this person who is simultaneously *a father*, *a policeman*, *a Māori*, *Jack*, *my husband*, *your son*?) are greatly diversified across languages in regard to semantics, combinability, grammatical status, and their role in linguistic and cultural practices.

Within the domain of human reference, one fundamental choice is whether or not to employ a kinship term. Every language has words for kin. These typically emerge early in a child’s linguistic development [2]. Kin terms are inherently relational; one cannot be a mother without having a child. The use of kinship terms highlights this relationality as the key to formulating reference to others: a person is identified through one or several other persons (e.g., English *Mary’s mother,* Dalabon *yengkulngu* ‘boy who is the nephew of one of us and the son of the other (we being opposite-sex siblings)’, [3]) rather than through an independent characteristic they possess as an individual (e.g., *woman, policeman, tall person, Mary*). Kinship terms are thus central to sociality, with the power to define, track, regulate and propagate networks of relationships (cf. [4]).

Do all cultures, then, formulate reference to others equally often in terms of kinship? We know there is massive variation in the meaning, complexity, and formal expression of kinship term sets across languages [5], but we know little about what influences the frequency of kinship mentions in spontaneous language use [6], nor what affects the prevalence of kinship as opposed to other formulation strategies in human reference. Here, we test factors that potentially associate with the frequency of kinship formulations in discourse (as compared with other human reference expressions), and we do so across 22 diverse languages.

In particular, we put the spotlight on a central question of diversity and dynamicity in language, cognition, and culture: is the usage frequency of terms from a semantic domain (here: kinship) associated with the integration of this domain into the grammar? For the kinship domain, we operationalise this by measuring *kintax* [7,8], the use of morphosyntactic means for encoding kinship in grammar. These include, for instance, special kinds of pronouns or agreement marking [9,10] for particular types of kin relationship, or dyadic markers that derive expressions with meanings like ‘mother and child’ from a noun meaning ‘mother’ or ‘child’ [11]. Although all 22 languages in our sample have kinship terms, only fifteen have some form of kintax as part of their grammar.

Unravelling the relationship between frequency of use of a semantic domain and its presence in grammar is central to understanding three key questions. (a) Why are the world’s 7,000+ languages so strikingly diverse? (b) What are the consequences of this diversity for how they are used and learned? (c) How do use and learning interact with the emergence of different grammatical structures? [12–16] Previous studies suggest that frequency of use is associated with the grammaticalisation of lexical items to grammatical elements (e.g., [17,18]), as well as being a factor in the elaboration of lexical distinctions [6,19] and, specifically, the velocity of lexical change within the kinship domain [20].

However, the causal relations are complex (cf. [21]), and the vast majority of the world’s languages lack accessible historical data. This makes it difficult to compare causal trajectories which might show whether greater frequency of use leads to more extensive grammaticalisation (here: greater frequency of kinship formulation leading to the development of kintax) or vice-versa. At this stage we address the first step in investigating questions of causality, namely determining whether any statistical association can be found. Matched contemporary data allow us to measure if the presence of certain grammatical features is a statistical predictor of whether kinship formulation is employed more frequently in the languages that have them. The question we thus ask in this article is whether there is a demonstrable relationship between the grammatical encoding of a semantic domain and how often that domain is evoked in spontaneous discourse. In the discussion, we delve into the direction of causality in such a relationship.

A serious methodological barrier to demonstrating an association between frequency of use and grammatical differences has been the lack of language corpora which are cross-linguistically comparable but which are unbiased by text founder effects. These text founder effects arise in parallel translation corpora (e.g., Bible translations), because the source language is in a privileged position in terms of which semantic categories get expressed, distorting the frequency of occurrence in the target languages. The reverse, comparing dissimilar corpora across languages, can introduce spurious differences. For example, a corpus of marriage and inheritance jurisdictions in one language would probably show a higher proportion of kinship references than a corpus of gardening manuals in another language. This would not reflect cross-linguistic differences in kinship term frequency, but the effects that genre-skewed sampling can have on virtually any semantic domain. Spontaneous conversation can avoid these pitfalls (e.g., [22,23]), but may be impenetrable in regard to features such as human reference without intimate knowledge of the real-life context. To overcome these barriers while ensuring comparability across languages, ‘parallax’ corpora [24] elicit spontaneous natural speech and signing across a range of languages in response to a common, constrained set of picture card stimuli, while allowing participants to choose their own ways of talking, without translation bias. Here we investigate the use of kin- versus non-kin-based formulations in human reference when people are presented with the same stimuli, using the Family Problems Picture Task: two participants describe a series of 16 picture-cards, negotiate to put them into a sequential order, and then narrate them as a story to a third (naïve) participant [25].

**Fig 1:**
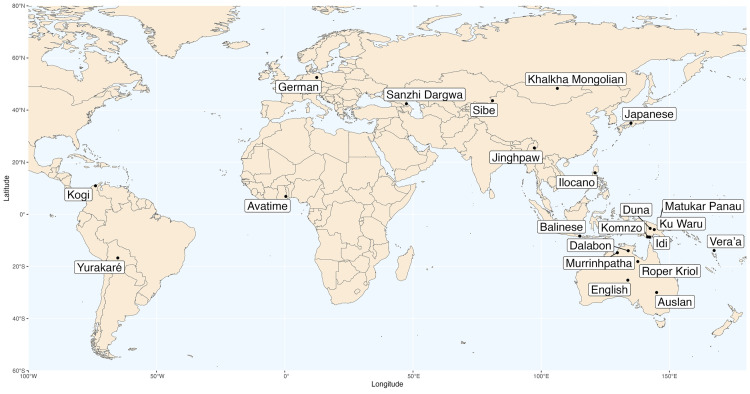
Map of approximate locations where data were collected. The map was created with the R packages rnaturalearth [26] and rnaturalearthdata [27], which draw on public domain data from Natural Earth.

In our comparison, we include data from 21 spoken languages and one signed language. The languages come from all over the world (see Fig 1 for a map of the languages’ locations) and from 16 different language families, thus reducing the risk of biasing our results by repeated sampling from language families or areas (see also SI-2.5 in S1 Appendix). Table 1 includes the languages’ names (and their ISO codes), language clades (families), countries, macro-regions, and details of which researchers collected and annotated the data.

**Table 1:** Languages in the present study.

Language	ISO code	Language clade	Country	Macro-region	Researcher(s)
**Auslan**	ASF	British Sign Language	Australia	Pacific	Gabrielle Hodge
**Avatime**	AVN	Niger-Congo	Ghana	Africa	Saskia van Putten
**Balinese**	BAN	Austronesian	Indonesia	Pacific	Wayan Arka
**Dalabon**	DAL	Australian	Australia	Australia	Nicholas Evans
**Duna**	DUC	Trans New Guinea	Papua New Guinea (PNG)	Pacific	Lila San Roque
**English**	ENG	Indo-European	Australia	Europe/Australia	Barb Kelly
**German**	DEU	Indo-European	Germany	Europe	Andrea C. Schalley
**Idi**	IDI	Pahoturi	PNG	Pacific	Volker Gast
**Ilocano**	ILO	Austronesian	Philippines	Pacific	Yukinori Kimoto
**Japanese**	JPN	Japonic	Japan	Pacific	Chikako Senge, Eri Kashima, Heiko Narrog & Nicholas Evans
**Jinghpaw**	KAC	Sino-Tibetan	China, India & Myanmar	Asia	Keita Kurabe
**Khalkha Mongolian**	MON	Mongolic	Mongolia	Asia	Dolgor Guntsetseg & Elena Skribnik
**Kogi**	KOG	Chibchan	Colombia	South America	Henrik Bergqvist & Dominique Knuchel
**Komnzo**	KOMNZO	Yam	PNG	Pacific	Christian Döhler
**Ku Waru**	MUX	Trans New Guinea	PNG	Pacific	Alan Rumsey
**Matukar Panau**	MJK	Austronesian	PNG	Pacific	Danielle Barth
**Murrinhpatha**	MWF	Australian	Australia	Australia	John Mansfield
**Roper Kriol**	ROP	Creole	Australia	Australia	Gregory Dickson
**Sanzhi Dargwa**	DAR	Nakh-Daghestanian	Russia	Europe	Diana Forker
**Sibe**	SJO	Tungusic	China	Asia	Norikazu Kogura
**Vera’a**	VRA	Austronesian	Vanuatu	Pacific	Stefan Schnell
**Yurakaré**	YUZ	Language isolate	Bolivia	South America	Sonja Gipper

**Fig 2:**
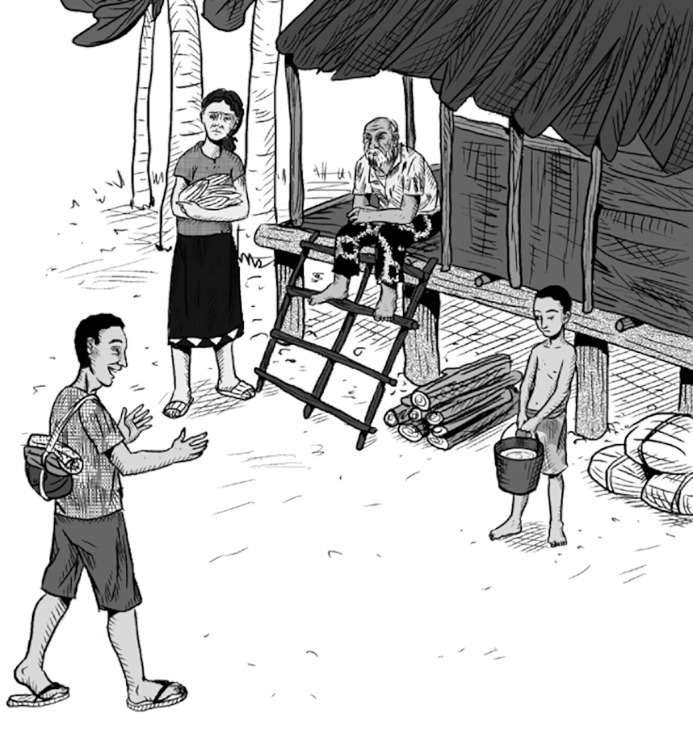
‘Homecoming’ card, stimuli example. (The figure is in the public domain and available at https://scopicproject.wordpress.com/run-the-task/ and https://scopicproject.wordpress.com/wp-content/uploads/2018/01/stimuli-pictures.pdf).

Since each language in the study has a range of terms available for talking about people, including kinship terms (*aunt*), generic terms (*woman*), role nouns (*policewoman*) and names (*Maria*), the choice of reference at any given moment is not forced by the task, but remains each participant’s (conscious or unconscious) decision. At the same time, by hypothesis, users of different languages may weight these choices in different ways. As an initial (and typical) illustration, contrast the examples from Kogi in (1) and German in (2), which both describe the first card of the task, reproduced in Fig 2.

Example (1) shows a kin-rich string of utterances from Kogi: every human character is identified through a kinship term. The speaker first describes the old man character as a grandfather, reckoning his identity from the perspective of the child character; then identifies the young man as a son in relation to the grandfather; and then switches perspective again to that of the young man, as he refines his assessment of their relationship, before identifying the young woman as a relative (the wife) of the young man.

(Examples in this paper are displayed using interlinear glossing; see at the end for a list of abbreviations used. Information in square brackets indicates the recording of the picture task and the time interval of the example. The recordings are available in [28].)

[1] *h*ẽ*ki    hate-dweba      h*ẽ*ki   a-skwá*
[2]  dem=foc  grandfather-old      dem=foc  3sg.poss-son
[3]  *ezhi    a-hwäsgwi        hálde=ki  ahí      munzhi*
[4]  or     3sg.poss-father.in.law   dem=foc   3sg.poss    woman/wife

‘This is the grandfather. This is his [the old man’s] son. Or his [the young man’s] father in law. This one is his [the young man’s] wife.’

[SocCog_kog01-CNC_130619_1 - 00:00:11-00:00:18]

Example (2) illustrates a German language description of the same scene that uses common nouns, descriptors for age differences and a role (*dieser Besuch* ‘this visitor’) rather than kin terms. The speaker relates characters to each other through comparative age, and no family ties are made explicit.

(2) *Da    drauf     sind    ein     älterer   Mann*
med    on.top      be.prs.pl  indf.sg.m   elderly.sg.m man
*eine    Frau     ein     Kind     und   dieser    Besuch,*
indf.sg.f    woman    indf.sg.n  child     and   dem.sg.m   visitor
*ein     junger    Mann*
indf.sg.m   young.sg.m     man

‘On it (the picture) are an elderly man, a woman, a child, and this visitor, a young man.’

[SocCog-deu01-hs_ks_HR_RS_PV2019-10-30 - 00:00:42-00:00:47]

The lexical options for human reference across languages are broadly comparable. For example, both German and Kogi have kin and non-kin nouns available in the lexicon, but the speakers of (1) and (2) made different choices about when to deploy which. We examine whether particular patterns of semantic construal (for example, whether one says *the father* as opposed to *the man*) are associated with the presence of grammatical complexity within the semantic domain of kinship. If grammatical structure is independent of the frequency with which words in the same semantic domain are used, we will not observe a relationship between the presence of kintax and the prevalence of kin-based formulations in our sample. However, if grammatical structure directly relates to formulation frequency, we expect to see statistical advantages from including kintax as a predictor in our model.

Having a multilingual corpus with many occurrences of human reference allows us to examine a preponderance of evidence: for each instance of reference to a human, we assess the factors that may influence whether the token is a kinship word or not. We build a generalized linear statistical model to show which factors matter most often in determining this (subconscious or conscious) choice by language users. In addition to kintax, we examine three further variables as possible predictors of kinship expression in the data, described and motivated in more detail in the section ‘Candidate predictors of kin term frequency and statistical model details’ and S1 Appendix SI-2: complexity of a language’s sibling-term system, discourse type (conversation vs narration), and scene content (type of picture-card being viewed, e.g., solitary character vs intergenerational group of characters). Evaluating the contribution of these factors helps to identify other potential influences on reference practice, and gives an indication as to what other features may regulate any observed relationship between grammaticalisation and lexical use frequency.

## Materials and methods

### The corpus

This study uses an open-ended multilingual corpus annotated with functional categories relevant to social cognition [24,28,29]. Languages were included in the corpus on the basis of having grammatical characteristics relevant to several facets of social cognition. To create the corpus, a stimulus-elicitation methodology combined with a narrative problem-solving task in a language documentation context was used [25], with the different task phases generating both broadly comparable conversational and narrative data across multiple languages. Ethical approval for this study was obtained in compliance with national and institutional regulations, and informed consent was gathered from all participants in either written or verbal form, in line with local conventions and practices. (The following formal approvals were granted: Australian National University Human Research Ethics Committee, approvals ANU 2008/253, ANU 2010/238, ANU 2013/055, ANU 2014/224, ANU 2015/768; Griffith University Human Research Ethics Committee, approval LAL/09/10/HCREC; La Trobe University Human Research Ethics Committee, approval LTU E15/68; University of Melbourne Human Research Ethics Committee, approval 1544741. The recruitment period for this study was 01/02/2009–31/10/2021. Additional information regarding the ethical, cultural, and scientific considerations specific to inclusivity in global research is included in the Supporting Information (S2 File).) Participants choose their own formulations for the same depicted situations, without influence from a source language (in contrast to translation tasks). The task results in interactions of around 20–60 minutes per group of speakers or signers: conversation in pairs (description and ordering of pictures) as well as, with an additional naïve participant to reset common knowledge, narrative story-telling. In a few circumstances, alternative versions of the task were run due to participants choosing to combine the description and ordering phases, or due to unavailability of an additional third participant. As the data collected are still highly comparable, we kept all versions.

The nature of the task is likely to affect the kinds of human reference structures that are used. For instance, participants generally talk about characters from the picture card stimuli in the third person, and they talk about characters new to them whom they can assume are also new to their interlocutors. This is likely to result in less use of kin terms that are anchored to the current speaker/signer or addressee (e.g., *mum*, *your brother*), and less use of proper names. However, it is quite clear through the distribution of reference terms in our data that many participants spontaneously and readily assigned kinship roles and relationships to the depicted characters. Very few participants used proper names; we take this to mean that kinship relationships are more readily generalizable and projectable to new referents than names.

### The languages and their features

Linguists with expertise in the relevant languages coded the corpus data for the presence of lexical noun-phrase reference to humans, and each instance was annotated as a kin or non-kin expression in ELAN [30,31] (*n* = 18,841 tokens after exclusions: datapoints from participants who produced less than four lexical human referent terms during their session were removed – these were usually tokens from the third naïve participant who was present for the latter phase of the task). Overall, more than half (53%) of the references to humans are carried out with kinship terms. Token numbers by language vary from 55 (Idi – one collected run of the task) to 5,319 (Balinese – 25 collected runs of the task). This variability is a feature of documentary data collection, where access to participants is often limited, expensive and dependent on the kinds of data that communities want to collect. Especially for under-described languages, we see even limited data points as a contribution to our knowledge of different languages and communities, so they are included in our analysis.

Table 2 shows the numeric and proportional uses of kin terms in the corpus data for each language, along with a measure of the structural (kintax) and lexical (sibling term complexity) designation for each language (see the section ‘Candidate predictors of kin term frequency and statistical model details’ for a discussion).

**Table 2:** Kin term usage by language (ordered by proportion of reference) and measures of language-dependent predictors tested. (Kin proportion calculated based on total terms, not as average of languages' kin proportions).

Language (ISO code)	Total terms	Kin-based formulations	Kin proportion	Kintax score	Sibling term complexity
**Ilocano (ILO)**	87	64	0.74	3	2
**Dalabon (DAL)**	316	219	0.69	4	4
**Balinese (BAN)**	5319	3608	0.68	2	3
**Kogi (KOG)**	344	216	0.63	2	4
**Jinghpaw (KAC)**	754	453	0.60	1	3
**Yurakaré (YUZ)**	2039	1200	0.59	1	6
**Matukar Panau (MJK)**	1652	910	0.55	2	3
**Sanzhi Dargwa (DAR)**	597	320	0.54	0	2
**Roper Kriol (ROP)**	282	151	0.54	1	2
**Vera’a (VRA)**	367	195	0.53	2	3
**Khalkha Mongolian (MON)**	2446	1264	0.52	2	3
**Avatime (AVN)**	557	286	0.51	1	4
**Sibe (SJO)**	192	97	0.51	1	4
**German (DEU)**	407	193	0.47	0	2
**Japanese (JPN)**	435	181	0.42	1	4
**Murrinhpatha (MWF)**	158	62	0.39	2	2
**English (ENG)**	553	211	0.38	0	2
**Idi (IDI)**	55	18	0.33	0	2
**Komnzo (KOMNZO)**	278	77	0.28	0	2
**Duna (DUC)**	219	48	0.22	0	3
**Auslan (ASF)**	1478	238	0.16	0	2
**Ku Waru (MUX)**	306	49	0.16	1	2
***Total terms/formulations***	*18841*	*10060*			
***Average proportion/score***			*0.53*	*1.18*	*2.91*

### Candidate predictors of kin term frequency and statistical model details

We examined four independent fixed-effects variables as predictors of the frequency of kinship formulation based on theories from the literature: 1) kintax, 2) complexity of a language’s sibling term system, 3) discourse type, and 4) content of the scene under discussion. We originally considered to also include a societal variable (society size or esotericity) and language-distributional variables (clade and macro-region), but had to exclude them for statistical reasons (see SI-2.5–SI-2.6 in S1 Appendix for more information).

Our goal was to determine if kintax contributes to kinship usage frequency even after taking into account alternative possibilities. Our methods for determining these variables are described in more detail in the four subsections of this section as well as in S1 Appendix SI–2.

In order to test the contribution of each factor, a hierarchical mixed-effects generalized linear (GLMM) regression model was produced using the packages {lme4} [32] and {lmerTest} [33] in *R* [34]. Using a multivariate approach allows for examining multiple potential influential factors simultaneously, and a mixed-effects approach permits us to distinguish random from fixed effects. We included random intercepts for four variables. We would expect changes to the intercepts if new datapoints were added to the study, but would expect the independent variables to still have a similar effect on new language data. First, a random intercept was included for language, because we consider our 22 included languages as a sample of the whole population of languages in the real world we could have collected data from. Participant group was included as a second random intercept and participants as a third random intercept, as we do not know *a priori* what effects participant group dynamics may have on the frequency of kinship-based formulations and if users of the same language use similar amounts of such formulations. We might expect different behaviour from new groups and users from our sampled language communities if they were to be added to the study. Therefore, a nested random effects structure was used with intercepts for language, participant group and participants. A fourth random intercept for the stimulus viewed at time of language production is included, as some stimuli may be more likely to generate kin-based formulations than others. The GLMM was logistic, as the dependent variable was binary: kinship formulation or another formulation for each lexical expression of human reference. We annotated each instance in our dataset for the four independent variables described below and then built the regression model to assess their contribution in predicting (being associated with) using a kinship or non-kinship term in human reference.

Unlike disciplines that run planned experiments over groups of people and have balanced designs, corpus linguistics uses data from naturalistic, and often spontaneous, linguistic behaviour. This means that the data are inevitably unbalanced. Some people produce many lexical referents, others few, so distribution of data across participants is uneven. Additionally, for our study, some languages are represented by more data points than others due to differing opportunities to collect data. A GLMM approach helps ameliorate the unbalanced nature of corpus data because the idiosyncrasies of the participant groups and languages are captured by the random effects and do not overly affect the regression coefficients (cf. [35]).

We now describe the four included variables in more detail.

#### Structural: Kintax score.

Grammatical consequences of using kinship terms may make language users more aware of kinship in their social world and therefore more likely to use kinship terms, or high levels of kinship term usage may have been the source for developing kintax, or both. We scored languages as having 0–5 categories of kintax based on whether these categories exist in the language or not: 1) obligatory possession of kinship terms, 2) special combinatorics for kinship terms, 3) in-group/out-group distinctions for kinship terms, 4) kin-based pronouns, and 5) kinship dyads. For descriptions of each category, see SI-2.1 in S1 Appendix.

#### Lexical: Sibling-term complexity.

It may be that users of languages with frequent mention of kinship are willing to ‘invest’ in a more complex kinship system, i.e., the more a semantic domain is used, the greater the payoffs for a more informative system [19]. We counted the number of sibling terms in a language as a proxy for overall kinship system complexity, where there is a maximum of eight possible terms using three features: i) gender of the sibling, ii) age of the sibling in relation to the speaker or signer and iii) gender of the speaker or signer (2x2x2). Other features are possible, but not common or directly comparable across our sample. The range of sibling terms in our sample is from 2 to 6 (see S1 Appendix SI-2.2 for a motivation, more details, and examples).

We now turn to variables that depend on task-specific contexts, rather than features of a language.

#### Interactional: Discourse type.

Tokens were coded as coming from either conversation (the first two phases of the task, where the two participants view, describe and arrange the cards together) or narrative (the third phase of the task, where the first two participants relate the story to a newcomer). In the conversational phase, the two participants must negotiate what the relationships are between the depicted characters (phase one) and establish a shared understanding of the story (phase two). In the narrative stage, they must introduce the (now mutually established) characters to another person. It may be that participants are more likely to present characters in stable relationships to other characters once they reach the narrative task phase and have constructed a fictional cast of characters, potentially increasing kinship reference in this part of the task.

#### Topical: Scene content and generational configuration.

We expect that context and the nature of the reference target contribute to reference formulation, and that in particular human character configurations of described events may be relevant in the context of this study. For the SCOPIC data, we use the depicted scene that language users are looking at and referring to at the time of speaking or signing. We classified these scenes into types based on the kind and number of human figures depicted in the stimuli (see SI-2.4 in S1 Appendix for more details): Solitary (only one character), Peer (characters appeared to be all of the same generation), Intergenerational (including either adults and a child or adults and a much older adult), and General (no specific card was being described/referred to by holding or looking at during the time of utterance).

## Results

**Fig 3:**
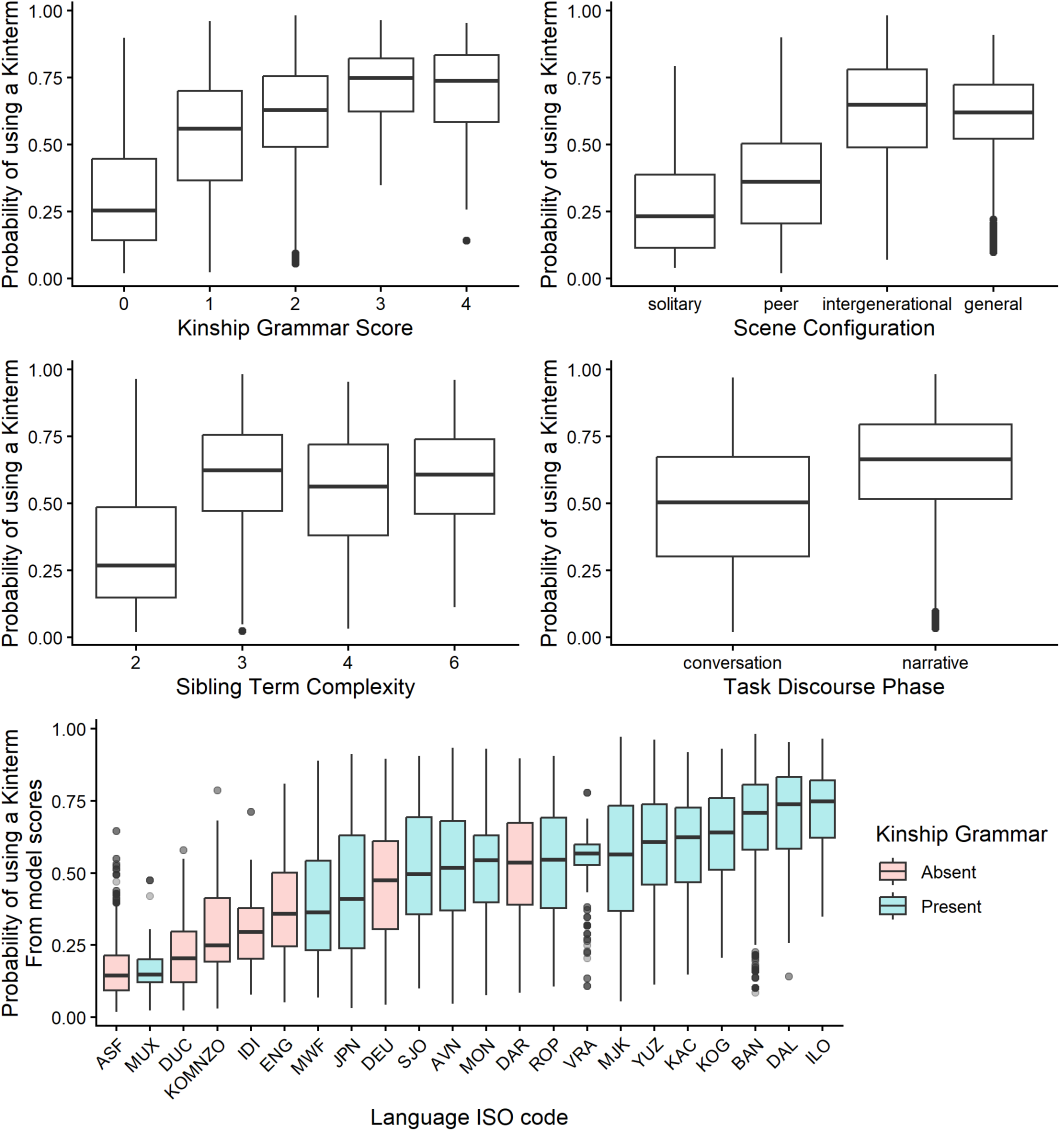
Kin-based formulations model probabilities by each predictor variable plus distributions for languages.

Fig 3 shows plots of the model probabilities for each predictor variable plus distributions for each language in our sample.

Our model has significant (at *p <* 0.05) predictors (fixed effects) due to structural kintax score (*Z* = 3.56, *p <* 0.001), but not due to sibling term lexical complexity (*Z* = 1.48, *p* = 0.14). Scenes with intergenerational configurations of people (*Z* = 3.69, *p <* 0.001) are more likely to be described or narrated with kinship terms than solitary depictions, showing that most task participants placed different-aged characters into kinship relationships with each other. The largest effect we see is from discourse type, where narrative phases of the task are much more likely to use kinship terms (*Z* = 13.87, *p* < 0.0001).

Our results clearly show that people who use languages which have more kinship-based grammar (kintax) use kinship terms more often than others, even taking into account other plausible predictors. Therefore, we can feel confident that having kintax is predictive of higher usage of kinship-based formulations in a language, establishing an empirical link between the possession of a grammatical structure and frequency of formulation.

Kintax is not the only determining factor, though: Narrative discourse was strongly associated with higher kinship reference frequency. We also see the frequency of kinship formulations being influenced by the number and kinds of characters visible in the picture stimuli. This suggests an influence of discourse type on reference patterns, and a general propensity for people to infer and express kin relationships in relation to depictions of co-present individuals, whereas solitary individuals are less likely to be put in relational configurations. We do not, however, see a significant link between the complexity of a language’s sibling term vocabulary and users’ frequency of kinship formulations. Therefore, while structural elaborateness in the kinship domain is a predictor for kinship formulation frequency, lexical elaborateness in this domain is not (given our operationalisations).

## Discussion

What is very clear from our results is that having at least some kinship grammar is associated with a higher frequency of use of kin-based formulations, and having more elaborated kintax is likewise associated with a higher rate of kin-based formulations (Fig 3). Thus lexical usage patterns in spontaneous discourse do correlate with structural patterns in grammar.

At this stage, it is not clear whether the relationship between having kintax and increased kinship word usage is causal nor, if it is, what the direction of causation would be. Does the presence of grammatical categories that pick out kinship make the concept of kinship more salient and more readily selected, a result of ‘thinking for speaking’ [36]? Or is it that the frequent choice to formulate reference in terms of kinship rather than some other way, iterated over many generations, drives the emergence of kintax – that ‘grammar is restless and earned’ ([37], p. 60) and that ‘grammars code best what speakers do most’ ([38], p. 363)? There is also a third possibility: that some further variable, like the greater cultural importance of kinship, could influence both usage frequency and the emergence of kintax, with no direct causal relationship in either direction between frequency and kintax.

Proper evaluation of the first two causal scenarios would require longitudinal studies, unfolding over centuries or even millennia, for a good sample of languages. To evaluate the third, we would need some independent way of evaluating the cultural importance of kinship (cf. [39]), something that would require careful operationalisation and more fieldwork. Yet, we can suggest the potential importance of feedback loops (cf. [23]), with differential frequency driving the emergence of different grammars, which then in their turn influence language users’ choices of formulation. The first two causal scenarios might therefore reinforce each other.

Even more complex causal mechanisms might be at work, and we now mention two of these briefly.

Firstly, it is likely that the effects of formulation frequency are stochastic rather than absolute, and are path-dependent in the sense of interacting with other features of the language. For example, see [8] on the intricacies of how Murrinhpatha sibling-dual pronouns evolved, and [7] for arguments that kinship-sensitive pronouns may only develop when kin-dyad constructions combine with inclusory constructions [40], in which ‘me and dad’ is expressed as ‘we dad’ (i.e., ‘dad’ is included in the referent set of ‘we’); the conjunction of these two conditions is a far from common situation.

Secondly, we might also expect other discourse practices, such as the existence of special registers sensitive to kinship relations like ‘mother-in-law’ or ‘avoidance’ registers (e.g., [8,41,42]), to influence attention to kinship relations. However, social practices which look superficially similar may have quite different effects on usage frequency of kin-based formulations. For example, many societies have name taboos, and this can promote the use of kinship terms as an alternative to proper names, potentially accelerating the development of kintax (see [41] on Murrinhpatha and [43] on Yélî Dnye). However, Ku Waru, which also has name taboos, has very low rates of kinship formulation and kintax. A possible reason is that Ku Waru has a different solution to the collision of name taboos with the challenge of how to clearly refer to others, namely the use of ‘food names’. ‘Food names’ link pairs of individuals based on some event where they shared a food item, so that one may say, e.g., ‘your banana’ in place of ‘your uncle’, thus allowing clear reference without invoking kin. Although there are no uses of these ‘food names’ in the Ku Waru picture task data, the fact that they are frequently used in everyday life by Ku Waru people in place of kin terms may be relevant to the otherwise unexpectedly low use of kin terms in our Ku Waru corpus, where, instead, generic referents such as words meaning ‘man’ or ‘woman’ are frequent (see also, e.g., [44,45] on other New Guinea groups). Such particularistic cultural taboos may thus affect usage frequency, and underline the importance of distinguishing the degree to which kinship is an organising factor in daily life (a fact about culture) from the frequency with which kinship relations are mentioned in talk (a fact about discourse).

Increasingly, the diversity of the world’s languages is seen not just as a kaleidoscopic reshuffling of the same factors, but as the result of numerous selection pressures, varying across language communities, that differentially favour the emergence of certain linguistic phenomena in certain language communities [46–49]. These factors may be genetic (e.g., palate shape), climatic (e.g., altitude or humidity), demographic (e.g., population size, proportion and influence of mother-tongue users vs immigrants), but also cultural, as we have started to show here via a cross-linguistic study of human reference. Cultural influence operates by favouring different patterns of discourse, themselves reflecting different cultural conventions in how humans talk about the world, then generating frequency patterns which eventually precipitate into grammatical differences. The methods employed in this article show us that deep differences exist in how members of different cultures formulate their talk about the social world, and that this correlates significantly with which grammatical structures are found.

## Interlinear glossing

The first line in language examples (such as (1) from Kogi) is a transcription in the orthography of the language, with hyphens to show breaks between morphemes (word parts). The gloss in the second line provides information about the meaning of each morpheme. The final part of the example is a translation into English. Abbreviations used in the glosses are:

dem  demonstrative

f   feminine gender

foc  focus marker

indf  indefinite

m   masculine gender

med  mediative

n   neuter gender

pl   plural

poss  possessive

prs  present tense

sg   singular

## Supporting information

S1 Appendix Data description, predictor and model building details, stimulus material. (PDF)

S2 File Inclusivity in global research. (DOCX)
