# Correction: Word choice is coupled with grammar: The case of kinship

**DOI:** 10.1371/journal.pone.0359296

**Published:** 2026-09-25

**Authors:** Danielle Barth, Nicholas Evans, Andrea C. Schalley, Lila San Roque, Sonja Gipper, John Mansfield, Gabrielle Hodge, Alan Rumsey, I. Wayan Arka, Henrik Bergqvist, Gregory Dickson, Christian Döhler, Diana Forker, Volker Gast, Dolgor Guntsetseg, Eri Kashima, Yukinori Kimoto, Dominique Knuchel, Norikazu Kogura, Keita Kurabe, Heiko Narrog, Desak Putu Eka Pratiwi, Stefan Schnell, Chikako Senge, Elena Skribnik, Saskia van Putten

In the Introduction section, there is an error in the 11th paragraph. The correct paragraph is: (Examples in this paper are displayed using interlinear glossing; see at the end for a list of abbreviations used. Information in square brackets indicates the recording of the picture task and the time interval of the example. The recordings are available in [28].)

(1) *hẽki hate-dweba hẽki a-skwá*

dem=foc grandfather-old dem=foc 3sg.poss-son

*ezhi a-hwäsgwi hálde=ki ahí munzhi*

or 3sg.poss -father.in.law dem=foc 3sg.poss woman/wife

‘This is the grandfather. This is his [the old man’s] son. Or his [the young man’s] father in law. This one is his [the young man’s] wife.’

[SocCog_kog01-CNC_130619_1 - 00:00:11-00:00:18]

The ORCID iDs are missing for the sixth, eighth, ninth, 12th, 13th, 17th, 18th, 19th, 21st and 25th authors. Please see the authors’ respective ORCID iDs here:

- Author John Mansfield’s ORCID iD is: 0000-0003-1167-1136 (https://orcid.org/0000-0003-1167-1136).
- Author Alan Rumsey’s ORCID iD is: 0000-0002-9710-4695 (https://orcid.org/0000-0002-9710-4695).
- Author Wayan Arka’s ORCID iD is: 0000-0002-2819-6186 (https://orcid.org/0000-0002-2819-6186)
- Author Christian Döhler’s ORCID iD is: 0000-0002-9659-5920 (https://orcid.org/0000-0002-9659-5920)
- Author Diana Forker’s ORCID iD is: 0000-0003-4247-9163 (https://orcid.org/0000-0003-4247-9163)
- Author Yukinori Kimoto’s ORCID iD is: 0009-0007-2505-5074 (https://orcid.org/0009-0007-2505-5074)
- Author Dominique Knuchel’s ORCID iD is: 0000-0003-1319-5786 (https://orcid.org/0000-0003-1319-5786)
- Author Norikazu Kogura’s ORCID iD is: 0000-0002-7163-5818 (https://orcid.org/0000-0002-7163-5818)
- Author Heiko Narrog’s ORCID iD is: 0000-0002-7436-0354 (https://orcid.org/0000-0002-7436-0354)
- Author Elena Skribnik’s ORCID iD is: 0000-0002-9673-2163 (https://orcid.org/0000-0002-9673-2163)
