# Supplementary material for: Word choice is coupled with grammar: The case of kinship
Source: PLoS One. 2026 Aug 14;21(8):e0355784. doi: 10.1371/journal.pone.0355784 (PMC13475811; doi:10.1371/journal.pone.0355784)
Supplement: Supplementary file 1 [file pone.0355784.s001.pdf]

Word choice is coupled with grammar: The case of kinship

S1 Appendix. Data description, predictor and model building details, stimulus material

The supporting information in this document is organised into the following sections:

SI-1 Data description
SI-2 Predictor details
SI-3. Model building details
SI-4. Stimulus Materials
SI-5. SI References

Dataset and R code for this project can be found in the 'Kinship based formulations typology' repository at https://osf.io/gh69y

SI-1 Data description

Table S1 below shows the rates of kin-based formulations by language, session group and individuals. Some Group IDs have only one individual as part of the group. This is due to either 1) low token production by other individual(s) in the group, and therefore the exclusion of those data points or 2) only one person was a participant in the group due to scarcity of people to participate in the task (a consideration that is unfortunately necessary in some endangered language documentation situations).

Table S1. Kin-based formulation usage by language, participant group and individual speakers and signers

Language (ISO code)	Total terms	Kin proportion	Group ID	Total terms (Group)	Kin proportion (Group)	Individuals ID	Total terms (Individual within Group)	Kin proportion (Individual within Group)
ASF	1478	0.16	ASF01	353	0.14	ASF_1	131	0.13
ASF	1478	0.16	ASF01	353	0.14	ASF_2	222	0.15
ASF	1478	0.16	ASF02	187	0.13	ASF_3	99	0.13
ASF	1478	0.16	ASF02	187	0.13	ASF_4	88	0.13
ASF	1478	0.16	ASF03	383	0.15	ASF_5	245	0.16
ASF	1478	0.16	ASF03	383	0.15	ASF_6	138	0.12
ASF	1478	0.16	ASF04	219	0.21	ASF_7	97	0.19

ASF	1478	0.16	ASF04	219	0.21	ASF_8	122	0.23
ASF	1478	0.16	ASF05	336	0.18	ASF_10	183	0.21
ASF	1478	0.16	ASF05	336	0.18	ASF_9	153	0.14
AVN	557	0.51	AVN01	203	0.67	AVN_01	154	0.70
AVN	557	0.51	AVN01	203	0.67	AVN_02	49	0.59
AVN	557	0.51	AVN02	89	0.49	AVN_04	6	0.33
AVN	557	0.51	AVN02	89	0.49	AVN_05	36	0.31
AVN	557	0.51	AVN02	89	0.49	AVN_06	47	0.66
AVN	557	0.51	AVN04	125	0.43	AVN_09	33	0.33
AVN	557	0.51	AVN04	125	0.43	AVN_11	92	0.47
AVN	557	0.51	AVN05	140	0.36	AVN_08	86	0.30
AVN	557	0.51	AVN05	140	0.36	AVN_10	54	0.46
BAN	5319	0.68	BAN01	213	0.60	BAN_01	213	0.60
BAN	5319	0.68	BAN02	255	0.60	BAN_02	255	0.60
BAN	5319	0.68	BAN03	157	0.75	BAN_03	157	0.75
BAN	5319	0.68	BAN04	314	0.69	BAN_04	314	0.69
BAN	5319	0.68	BAN05	232	0.44	BAN_05	232	0.44
BAN	5319	0.68	BAN06	147	0.70	BAN_06	147	0.70
BAN	5319	0.68	BAN07	287	0.56	BAN_07	287	0.56
BAN	5319	0.68	BAN08	59	0.75	BAN_08	59	0.75
BAN	5319	0.68	BAN09	284	0.55	BAN_09	284	0.55
BAN	5319	0.68	BAN10	213	0.44	BAN_10	213	0.44
BAN	5319	0.68	BAN11	336	0.57	BAN_11	336	0.57
BAN	5319	0.68	BAN12	286	0.66	BAN_12	286	0.66
BAN	5319	0.68	BAN13	303	0.75	BAN_13	303	0.75
BAN	5319	0.68	BAN14	263	0.72	BAN_56	48	0.81
BAN	5319	0.68	BAN14	263	0.72	BAN_57	141	0.62
BAN	5319	0.68	BAN14	263	0.72	BAN_58	74	0.84
BAN	5319	0.68	BAN15	289	0.72	BAN_59	162	0.72
BAN	5319	0.68	BAN15	289	0.72	BAN_60	79	0.68
BAN	5319	0.68	BAN15	289	0.72	BAN_61	48	0.77
BAN	5319	0.68	BAN16	250	0.77	BAN_62	141	0.79
BAN	5319	0.68	BAN16	250	0.77	BAN_63	59	0.71
BAN	5319	0.68	BAN16	250	0.77	BAN_64	50	0.80
BAN	5319	0.68	BAN17	257	0.82	BAN_65	94	0.84
BAN	5319	0.68	BAN17	257	0.82	BAN_66	139	0.79
BAN	5319	0.68	BAN17	257	0.82	BAN_67	24	0.96
BAN	5319	0.68	BAN18	135	0.81	BAN_68	92	0.78
BAN	5319	0.68	BAN18	135	0.81	BAN_69	16	0.88
BAN	5319	0.68	BAN18	135	0.81	BAN_70	27	0.85
BAN	5319	0.68	BAN19	204	0.76	BAN_71	55	0.82
BAN	5319	0.68	BAN19	204	0.76	BAN_72	123	0.70
BAN	5319	0.68	BAN19	204	0.76	BAN_73	26	0.92
BAN	5319	0.68	BAN20	167	0.78	BAN_74	69	0.74

BAN	5319	0.68	BAN20	167	0.78	BAN_75	60	0.80
BAN	5319	0.68	BAN20	167	0.78	BAN_76	38	0.84
BAN	5319	0.68	BAN21	129	0.66	BAN_77	46	0.70
BAN	5319	0.68	BAN21	129	0.66	BAN_78	54	0.50
BAN	5319	0.68	BAN21	129	0.66	BAN_79	29	0.90
BAN	5319	0.68	BAN22	111	0.83	BAN_80	25	0.80
BAN	5319	0.68	BAN22	111	0.83	BAN_81	52	0.81
BAN	5319	0.68	BAN22	111	0.83	BAN_82	34	0.88
BAN	5319	0.68	BAN23	209	0.75	BAN_83	136	0.71
BAN	5319	0.68	BAN23	209	0.75	BAN_84	45	0.82
BAN	5319	0.68	BAN23	209	0.75	BAN_85	28	0.82
BAN	5319	0.68	BAN24	124	0.90	BAN_86	35	0.94
BAN	5319	0.68	BAN24	124	0.90	BAN_87	34	0.85
BAN	5319	0.68	BAN24	124	0.90	BAN_88	55	0.91
BAN	5319	0.68	BAN25	95	0.88	BAN_88	32	0.72
BAN	5319	0.68	BAN25	95	0.88	BAN_89	34	0.97
BAN	5319	0.68	BAN25	95	0.88	BAN_90	29	0.97
DAL	316	0.69	DAL01	162	0.75	DAL_01	31	0.84
DAL	316	0.69	DAL01	162	0.75	DAL_02	131	0.73
DAL	316	0.69	DAL02	45	0.47	DAL_04	45	0.47
DAL	316	0.69	DAL03	109	0.70	DAL_05	109	0.70
DAR	597	0.54	DAR01	169	0.47	DAR_176	65	0.51
DAR	597	0.54	DAR01	169	0.47	DAR_177	104	0.45
DAR	597	0.54	DAR02	98	0.48	DAR_178	84	0.51
DAR	597	0.54	DAR02	98	0.48	DAR_179	14	0.29
DAR	597	0.54	DAR03	183	0.67	DAR_180	168	0.66
DAR	597	0.54	DAR03	183	0.67	DAR_181	15	0.73
DAR	597	0.54	DAR04	147	0.48	DAR_182	94	0.39
DAR	597	0.54	DAR04	147	0.48	DAR_183	53	0.64
DEU	407	0.47	DEU01	175	0.33	DEU_1	98	0.40
DEU	407	0.47	DEU01	175	0.33	DEU_2	77	0.25
DEU	407	0.47	DEU02	88	0.51	DEU_4	52	0.50
DEU	407	0.47	DEU02	88	0.51	DEU_5	25	0.44
DEU	407	0.47	DEU02	88	0.51	DEU_6	11	0.73
DEU	407	0.47	DEU03	144	0.63	DEU_07	72	0.50
DEU	407	0.47	DEU03	144	0.63	DEU_08	72	0.75
DUC	219	0.22	DUC01	58	0.31	DUC_01	50	0.30
DUC	219	0.22	DUC01	58	0.31	DUC_02	8	0.38
DUC	219	0.22	DUC03	161	0.19	DUC_07	132	0.19
DUC	219	0.22	DUC03	161	0.19	DUC_09	29	0.17
ENG	553	0.38	ENG01	130	0.32	ENG_07	76	0.34
ENG	553	0.38	ENG01	130	0.32	ENG_10	54	0.30
ENG	553	0.38	ENG02	120	0.35	ENG_03	58	0.19
ENG	553	0.38	ENG02	120	0.35	ENG_04	62	0.50

ENG	553	0.38	ENG03	99	0.36	ENG_01	43	0.40
ENG	553	0.38	ENG03	99	0.36	ENG_08	56	0.34
ENG	553	0.38	ENG04	100	0.48	ENG_06	53	0.38
ENG	553	0.38	ENG04	100	0.48	ENG_09	47	0.60
ENG	553	0.38	ENG05	81	0.47	ENG_02	44	0.55
ENG	553	0.38	ENG05	81	0.47	ENG_05	37	0.38
ENG	553	0.38	ENG06	23	0.22	ENG_11	14	0.14
ENG	553	0.38	ENG06	23	0.22	ENG_12	9	0.33
IDI	55	0.33	IDI01	55	0.33	IDI_1	55	0.33
ILO	87	0.74	ILO01	87	0.74	ILO_91	33	0.61
ILO	87	0.74	ILO01	87	0.74	ILO_92	54	0.81
JPN	435	0.42	JPN01	238	0.26	JPN_942	15	0.20
JPN	435	0.58	JPN01	238	0.74	JPN_943	6	1.00
JPN	435	0.42	JPN01	238	0.26	JPN_94	179	0.20
JPN	435	0.42	JPN01	238	0.26	JPN_95	38	0.58
JPN	435	0.42	JPN02	115	0.57	JPN_100	63	0.48
JPN	435	0.42	JPN02	115	0.57	JPN_991	7	0.43
JPN	435	0.42	JPN02	115	0.57	JPN_99	45	0.73
JPN	435	0.42	JPN03	82	0.66	JPN_31	29	0.59
JPN	435	0.42	JPN03	82	0.66	JPN_32	44	0.70
JPN	435	0.42	JPN03	82	0.66	JPN_33	9	0.67
KAC	754	0.60	KAC01	281	0.56	KAC_106	209	0.56
KAC	754	0.60	KAC01	281	0.56	KAC_107	72	0.53
KAC	754	0.60	KAC02	190	0.68	KAC_108	92	0.70
KAC	754	0.60	KAC02	190	0.68	KAC_109	98	0.66
KAC	754	0.60	KAC03	283	0.59	KAC_110	149	0.64
KAC	754	0.60	KAC03	283	0.59	KAC_111	134	0.54
KOG	344	0.63	KOG01	123	0.72	KOG_112	42	0.81
KOG	344	0.63	KOG01	123	0.72	KOG_113	81	0.68
KOG	344	0.63	KOG02	145	0.59	KOG_114	110	0.57
KOG	344	0.63	KOG02	145	0.59	KOG_115	35	0.63
KOG	344	0.63	KOG03	76	0.55	KOG_116	58	0.55
KOG	344	0.63	KOG03	76	0.55	KOG_117	18	0.56
KOMNZO	278	0.28	KOMNZO01	151	0.18	KOMNZO_04	111	0.18
KOMNZO	278	0.28	KOMNZO01	151	0.18	KOMNZO_05	40	0.18
KOMNZO	278	0.28	KOMNZO02	127	0.39	KOMNZO_03	74	0.35
KOMNZO	278	0.28	KOMNZO02	127	0.39	KOMNZO_1	53	0.45
MJK	1652	0.55	MJK01	236	0.46	MJK_122	127	0.41
MJK	1652	0.55	MJK01	236	0.46	MJK_123	109	0.52
MJK	1652	0.55	MJK02	286	0.46	MJK_124	152	0.47
MJK	1652	0.55	MJK02	286	0.46	MJK_125	134	0.46
MJK	1652	0.55	MJK03	146	0.63	MJK_127	81	0.72
MJK	1652	0.55	MJK03	146	0.63	MJK_128	55	0.62
MJK	1652	0.45	MJK03	146	0.37	MJK_129	10	1.00

MJK	1652	0.55	MJK04	114	0.40	MJK_130	71	0.51
MJK	1652	0.55	MJK04	114	0.40	MJK_131	43	0.23
MJK	1652	0.55	MJK05	43	0.79	MJK_132	36	0.75
MJK	1652	0.55	MJK05	43	0.79	MJK_133	7	1.00
MJK	1652	0.55	MJK06	169	0.80	MJK_134	100	0.79
MJK	1652	0.55	MJK06	169	0.80	MJK_135	62	0.84
MJK	1652	0.55	MJK06	169	0.80	MJK_136	7	0.71
MJK	1652	0.55	MJK07	187	0.73	MJK_137	112	0.76
MJK	1652	0.55	MJK07	187	0.73	MJK_138	60	0.70
MJK	1652	0.55	MJK07	187	0.73	MJK_139	15	0.67
MJK	1652	0.55	MJK08	150	0.43	MJK_140	28	0.11
MJK	1652	0.55	MJK08	150	0.43	MJK_141	64	0.45
MJK	1652	0.55	MJK08	150	0.43	MJK_142	58	0.57
MJK	1652	0.55	MJK09	198	0.39	MJK_143	134	0.49
MJK	1652	0.55	MJK09	198	0.39	MJK_144	64	0.20
MJK	1652	0.55	MJK10	123	0.66	MJK_146	83	0.66
MJK	1652	0.55	MJK10	123	0.66	MJK_147	40	0.65
MON	2446	0.52	MON01	299	0.52	MON_150	140	0.50
MON	2446	0.52	MON01	299	0.52	MON_151	159	0.53
MON	2446	0.52	MON03	182	0.49	MON_152	135	0.40
MON	2446	0.52	MON03	182	0.49	MON_153	47	0.74
MON	2446	0.52	MON04	123	0.39	MON_154	94	0.36
MON	2446	0.52	MON04	123	0.39	MON_155	29	0.48
MON	2446	0.52	MON05	446	0.59	MON_156	148	0.55
MON	2446	0.52	MON05	446	0.59	MON_157	298	0.60
MON	2446	0.52	MON06	311	0.56	MON_158	191	0.62
MON	2446	0.52	MON06	311	0.56	MON_159	120	0.46
MON	2446	0.52	MON07	277	0.67	MON_160	120	0.79
MON	2446	0.52	MON07	277	0.67	MON_161	157	0.58
MON	2446	0.52	MON08	251	0.49	MON_SG	91	0.44
MON	2446	0.52	MON08	251	0.49	MON_UA	160	0.51
MON	2446	0.52	MON09	221	0.41	MON_ES	66	0.44
MON	2446	0.52	MON09	221	0.41	MON_XX	155	0.40
MON	2446	0.52	MON10	336	0.41	MON_DE	207	0.42
MON	2446	0.52	MON10	336	0.41	MON_ZB	129	0.40
MUX	306	0.16	MUX01	306	0.16	MUX_02	21	0.14
MUX	306	0.16	MUX01	306	0.16	MUX_07	121	0.14
MUX	306	0.16	MUX01	306	0.16	MUX_08	164	0.18
MWF	158	0.39	MWF01	64	0.36	MWF_168	64	0.36
MWF	158	0.39	MWF02	36	0.61	MWF_170	15	0.53
MWF	158	0.39	MWF02	36	0.61	MWF_171	21	0.67
MWF	158	0.39	MWF03	58	0.29	MWF_172	36	0.33
MWF	158	0.39	MWF03	58	0.29	MWF_173	22	0.23
ROP	282	0.54	ROP01	112	0.45	ROP_174	112	0.45

ROP	282	0.54	ROP02	170	0.59	ROP_175	170	0.59
SJO	192	0.51	SJO01	192	0.51	SJO_01	102	0.57
SJO	192	0.51	SJO01	192	0.51	SJO_02	90	0.43
VRA	367	0.53	VRA01	270	0.54	VRA_01	141	0.54
VRA	367	0.53	VRA01	270	0.54	VRA_02	129	0.53
VRA	367	0.53	VRA02	97	0.52	VRA_03	71	0.46
VRA	367	0.53	VRA02	97	0.52	VRA_04	26	0.65
YUZ	2039	0.59	YUZ103	343	0.50	YUZ_1	284	0.45
YUZ	2039	0.59	YUZ103	343	0.50	YUZ_2	42	0.71
YUZ	2039	0.59	YUZ103	343	0.50	YUZ_3	17	0.82
YUZ	2039	0.59	YUZ104	297	0.62	YUZ_3	5	0.40
YUZ	2039	0.59	YUZ104	297	0.62	YUZ_4	217	0.63
YUZ	2039	0.59	YUZ104	297	0.62	YUZ_5	75	0.61
YUZ	2039	0.59	YUZ105	260	0.60	YUZ_3	15	0.67
YUZ	2039	0.59	YUZ105	260	0.60	YUZ_6	164	0.59
YUZ	2039	0.59	YUZ105	260	0.60	YUZ_7	81	0.63
YUZ	2039	0.59	YUZ106	362	0.59	YUZ_3	9	0.78
YUZ	2039	0.59	YUZ106	362	0.59	YUZ_8	223	0.66
YUZ	2039	0.59	YUZ106	362	0.59	YUZ_9	130	0.47
YUZ	2039	0.59	YUZ107	339	0.56	YUZ_10	200	0.49
YUZ	2039	0.59	YUZ107	339	0.56	YUZ_11	132	0.67
YUZ	2039	0.59	YUZ107	339	0.56	YUZ_3	7	0.57
YUZ	2039	0.59	YUZ108	241	0.72	YUZ_12	121	0.64
YUZ	2039	0.59	YUZ108	241	0.72	YUZ_13	106	0.78
YUZ	2039	0.59	YUZ108	241	0.72	YUZ_3	14	0.93
YUZ	2039	0.59	YUZ109	197	0.55	YUZ_14	146	0.49
YUZ	2039	0.59	YUZ109	197	0.55	YUZ_15	51	0.73

SI-2 Predictor details

Here, we describe additional details about the predictors (SI-2.1–SI-2.4), including a thorough account of the categories of ‘kintax’ for which we assessed each language. In addition, we discuss the societal and language-distributional variables we also considered but had to exclude for statistical reasons (SI-2.5–SI-2.6). We finish the section with a brief discussion of the dependent variable (SI-2.7).

SI-2.1 Structural: Kintax

Kintax is obligatory morphosyntactic encoding that is present when expressing kinship terms. We identified five categories of kintax that exist cross-linguistically: 1) obligatory possession with kinship terms, 2) special combinatorics for kinship terms, 3) in-group/out-

group distinctions for kinship terms, 4) kin-based pronouns, and 5) kinship dyads. Descriptions of each category are found below. Table S2 follows showing which languages have each of these categories.

Obligatory kin term possession refers to a situation where the use of a kin term entails a possessor, to the point where it is ungrammatical to say just “mother” or “the mother” as opposed to “my/your/…etc. mother”. For example, in Matukar Panau, the kin reference term *nen* ‘mother’ always co-occurs with a possessive pronoun that indicates the possessor as in *ham nen* ‘your mother’ or *ngahau nen* ‘my mother’. This contrasts with the general reference term *pain* ‘woman’, which does not need to be possessed. Obligatory kin term possession can involve additional complex restrictions and nuances. In Khalkha-Mongolian, which has obligatory possession for kin terms with concrete reference, first person plural possessor marking (*manaj/maan’* ‘our’) is often preferred over singular marking (*minij/min’* ‘my’), even if the possessor is literally just one person. This “plurality” can be explained by the way the family is perceived in the Mongolian culture: “I” am not alone, with respect to my family, and there is always a “we” implicated in any kinship relationship.

Cross-linguistically, obligatory possession most commonly involves body parts (which are naturally conceived of as part of something else) or kin terms (which logically cannot exist without a relationship between two persons). Obligatory possession not only helps to make referents identifiable, but also links them to their wider societal context [1].

Special combinatorics involve kin terms showing distinctive patterns of combination with other morphological or syntactic items.[1] A well-known example is Italian [2], in which possessives of non-kin expressions take articles in addition to possessive pronouns (*la mia casa* ‘my house’, literally ‘the my house’), whereas (consanguineal) kinship expressions take just the possessive pronoun without the article (*mia madre* ‘my mother’). Some examples in our data come from Dalabon, Khalkha Mongolian and Balinese. Dalabon speakers have the capacity to form a special verb by incorporating a kinship root into the formative -*ngandung*, yielding the meaning ‘X calls Y …’ , e.g. *bûkah-kardak-ngandung* ‘(s)he calls him uncle’ [3]. In Khalkha-Mongolian, kinship terms behave differently from common nouns in relation to accusative and reflexive inflection, as ‘[d]irect objects expressing a kinship relation must occur with an overt accusative case suffix’ ([4], p. 82). In Balinese, syntactic restrictions govern the possible interpretation of possession relationships: unmarked kin nouns —like body-part nouns ([5], p. 233)—are only understood as possessed when the person denoted by the kin term is an object, and the possessor is a subject

[1] Obligatory possession can be viewed as a specific kind of special combinatoric, but because it is such a prevalent one and one that affects large parts of the language system, we have considered it its own category for this analysis. Languages scored for having special combinatorics have other morphological or syntactic features specific to kinship.

In-group/out-group describes a situation where a language encodes additional distinctions in relation to whether the language user is referring to someone from their own in-group (typically, their family) or not. In our sample, this feature is found only in Japanese. Honorific markers in Japanese can be used to create distance (out-group, non-kin) or intimacy (in-group, kin) between the listener and speaker [6, 7]. This same paradigmatic distinction is also encoded in some kin terms themselves, for example, when referring to one's own wife or the wife of another family member, one uses *tsuma* 'wife.ingroup'. However, when referring to the wife of someone who is not related to the speaker, one uses *okusan* 'wife.outgroup'.

Kin pronouns are special pronominal forms that are sensitive to kinship relations between the referents designated by the pronoun. In our sample, this is found in Murrinhpatha [8, 9] and Dalabon [10]. For example, in Murrinpatha the prefix *nudde-* is used on a verb when exhorting two siblings to take action, encoding verbal modality, dual number, and the sibling relationship in a single grammatical form.

Kinship dyads are special grammatical means used to express particular paired (dyadic) relationships [11–13]. In languages with productive kinship dyads, an affix is attached to a noun to yield a noun K-DYAD meaning 'a pair of people, one of whom is K to the other', as in *mother-DYAD* meaning 'mother and child' or *wife-DYAD* meaning 'wife and husband'. An example in Dalabon is the dyad term *bey-ko* 'father and son pair or pair made up of a paternal aunt with her brother's son' (*bey* means 'male child through the male line').

Table S2. Kintax categories in each sample language

Language	Obligatory possession of kinship terms	Special combinatorics for kinship terms	In-group/out-group distinctions for kinship terms	Kin-based pronouns	Kinship dyads
**ASF**					
**AVN**		X			
**BAN**	X	X			
**DAL**	X	X		X	X
**DUC**					
**ENG**					
**DEU**					
**IDI**					
**ILO**	X	X			X
**JPN**			X		
**KAC**		X			
**MON**	X	X			

**KOG**	X	X			
**KOMNZO**					
**ROP**					X
**MUX**		X			
**MJK**	X	X			
**MWF**		X		X	
**DAR**					
**SJO**	X				
**VRA**	X	X			
**YUZ**	X				

SI-2.2 Lexical: Sibling term complexity

[14] argue that if a system has many different kinship terms, it has a high communicative cost for the speaker or signer (who has to select the right term from many choices), but is more informative (cf. [15–17] for kinship, *inter alia*), carrying a lower cognitive cost for the listener. According to [14], languages will be more likely to 'invest' in increased system complexity for domains which are frequently mentioned: the more often a domain is talked about, the greater the payoffs of a more informative system. Thus, the complexity of the kinship term system could be expected to correlate to kin term frequency.

[18] calculate kinship system complexity by a series of rules that are needed to distinguish all relations from each other and weigh all languages in their sample by the same need probability (i.e. probability of the need to use the term, based on corpus frequencies from English and German corpora). However, we cannot assume that the frequency of kinship usage is the same across our languages as this is what we attempt to test. Therefore, we turn to the domain of sibling terms. As discussed in the main text, our operationalisation of kinship term complexity is the number of sibling terms, scaled for standardisation. We have complete information for this semantic domain across our language sample (for some languages we have incomplete information for domains that can be harder to find out about: in-laws, third generation terms, etc., making these less useful cross-linguistic comparative domains). Sibling term complexity has been studied by other scholars such as [19].

The sibling category offers at least eight possible relationships depending on i) gender of the sibling, ii) age of the sibling in relation to the speaker or signer and iii) gender of the speaker or signer (2x2x2). A range of different patterns and complexity values are present in our sample. For example, German has two different sibling terms according to sibling gender, *Bruder* for a male sibling, and *Schwester* for a female sibling, giving a complexity score of 2. Kómnzo also has a score of 2, but here the two terms distinguish relative age rather than gender: *nane* for an elder sibling (male or female), and *ngth* for a younger sibling. Other

systems are more complex, but less ambiguous. The most complex system in our sample is that of Yurakaré ([20], p. 317), with six distinct terms: *pii* 'elder brother of male speaker', *pipi* 'elder brother of female speaker', *paa* 'younger brother of male speaker', *dyama* 'younger sibling of female speaker', *yee* 'sister of male speaker' and *neshu* 'elder sister of female speaker'.

There are additional components of sibling terms that can increase complexity, but these do not cut across all languages, and therefore are not part of our operationalisation. As an example, Matukar Panau has an additional sibling term *matu* for the eldest brother or an eldest uncle. We found that it was more cross-linguistically comparable to use our three-feature system to label the languages in our sample.

SI-2.3 Interactional: Discourse type

We tested whether the discourse type in the task made a difference to kin-based formulation frequency. In Fig S1 we see that for many languages their use of kin-based formulations was slightly higher in narrative contexts.

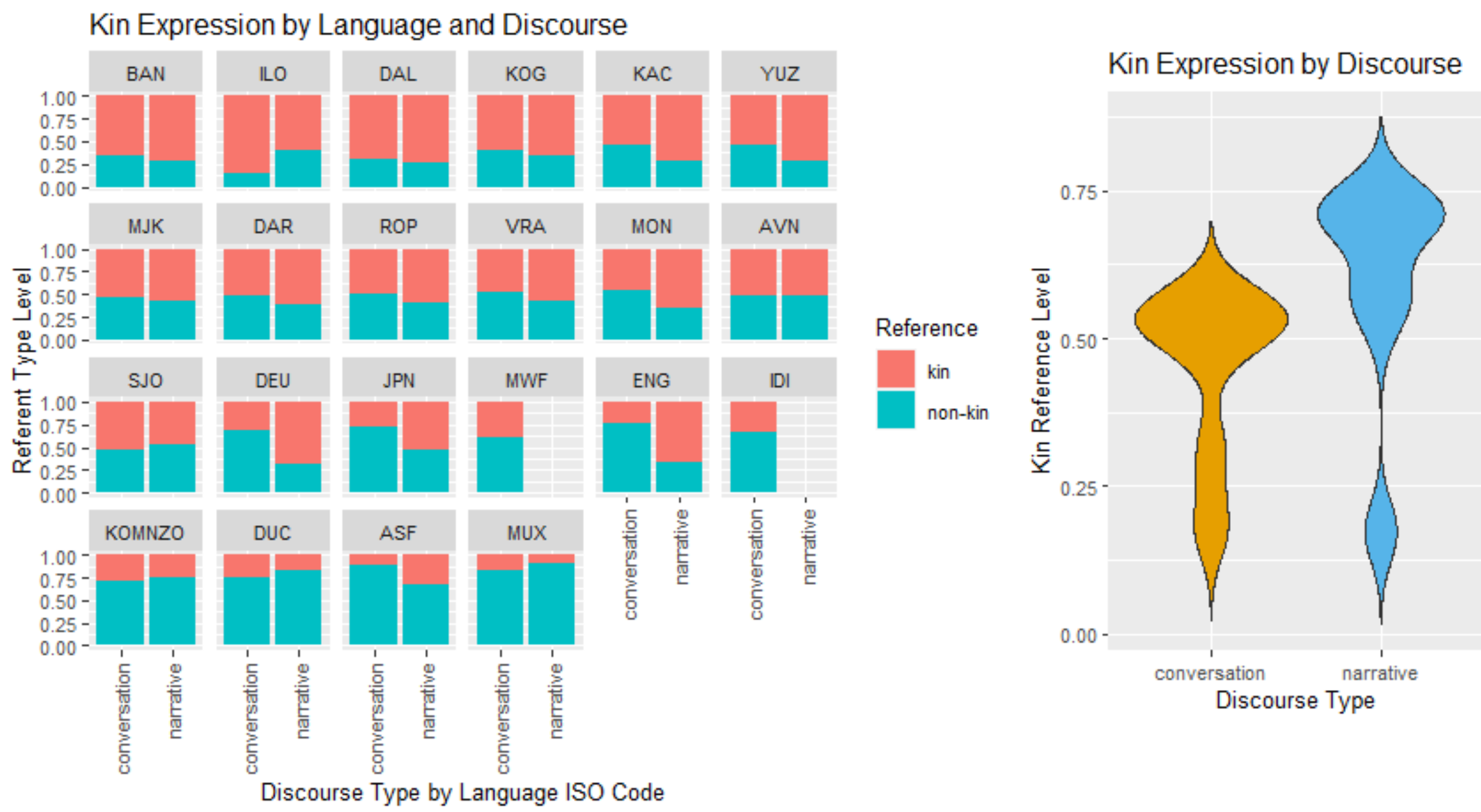

Fig S1. Kinship referents by language and discourse type

Note that for Idi (IDI) and Murrinhpatha (MWF), speakers combined the task phases and created a story as they described the cards, so that there was no clear distinction between a conversational phase and narrative phase. Table S3 presents the numbers of referents by task discourse type for each language with counts of kinship-based formulations in parentheses.

Table S3. Referent frequency (raw) by task discourse type and language

Language	Conversation	Narrative
**ASF**	1174 (138)	304 (100)
**AVN**	375 (194)	182 (92)
**BAN**	3013 (1967)	2306 (1641)
**DAL**	297 (205)	19 (14)
**DAR**	459 (235)	138 (85)
**DEU**	246 (80)	161 (113)
**DUC**	115 (30)	104 (18)
**ENG**	364 (84)	189 (127)
**IDI**	55 (18)	NA
**ILO**	48 (41)	39 (23)
**JPN**	196 (53)	239 (128)
**KAC**	502 (271)	252 (182)
**KOG**	154 (91)	190 (125)
**KOMNZO**	195 (57)	83 (20)
**MJK**	1113 (601)	539 (309)
**MON**	1777 (826)	669 (438)
**MUX**	248 (44)	58 (5)
**MWF**	158 (62)	NA
**ROP**	177 (88)	105 (63)
**SJO**	100 (53)	92 (44)
**VRA**	140 (66)	227 (129)
**YUZ**	1445 (774)	594 (426)

SI-2.4 Topical: Scene content and generational configuration

The configuration of the people depicted on each stimuli card was defined as: Solitary (only one character), Peer (characters appeared to be all of the same generation), and Intergenerational (including either adults and a child or adults and a much older adult). The classification of the cards is listed in Table S4. All data were annotated to show which of the 16 cards was being discussed at the time of each human referent expression. Tokens that were not associated with a particular card (e.g., they are produced as part of a more general discussion or while participants were not attending to a particular card) are combined into a level called "General". This provided us with a baseline for each language, used as the reference level for this predictor in our statistic model, allowing us to ascertain which kinds of scenes are associated with more or fewer kinship referents than general speech not associated with a particular scene.

Table S4. Card names and classification with kin-based formulation frequency (raw)

Card Name	Classification	Kin	Non-kin
1_Homecoming	Intergenerational	1034	527
2_Receiving clothes	Peer	87	468
3_Alone in the cell	Solitary	70	195
4_Drunken gossip	Peer	589	693
5_In court	Peer	460	841
6_Walking together	Intergenerational	584	424
7_Sitting drinking	Peer	328	582
8_Garden together	Peer	276	363
9_Taken by police	Intergenerational	576	730
10_Thinking about gaol	Peer	137	296
11_Refusing drink	Intergenerational	573	465
12_Hitting	Intergenerational	852	435
13_Family talking together	Intergenerational	681	305
14_Standing in light	Solitary	65	164
15_About to hit	Intergenerational	793	638
16_Thinking of home	Intergenerational	780	167
General/Non-Specific	General	2175	1488

The Peer and Solitary configurations have significantly fewer kinship expressions than the general or intergenerational contexts for many of the languages in our sample (see Fig S2).

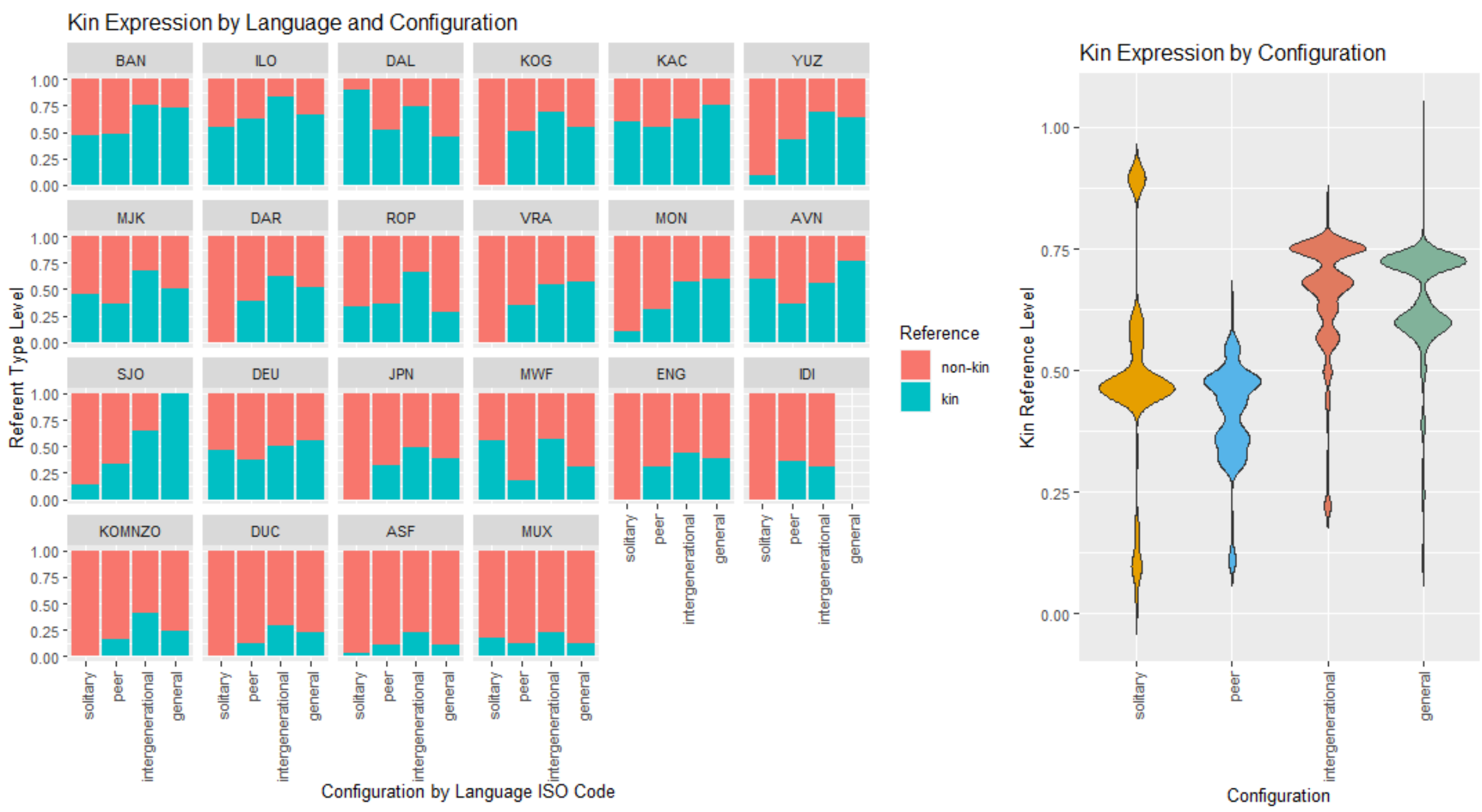

Fig S2. Kinship referents by language and scene content (generational configuration)

For Idi, 'General' in Fig S2 is empty because lexical human referents were only ever produced when specific cards were being discussed. Table S5 presents the numbers of referents by configuration for each language in the sample.

Table S5. Referent frequency (raw) by configuration and language

ISO	Solitary	Peer	Intergenerational	General
**ASF**	73	581	742	82
**AVN**	15	138	387	17
**BAN**	94	1198	2609	1418
**DAL**	19	74	212	11
**DAR**	13	182	369	33
**DEU**	13	105	256	33
**DUC**	11	68	123	17
**ENG**	16	172	293	72
**IDI**	1	22	32	0
**ILO**	11	19	48	9
**JPN**	8	140	233	54
**KAC**	5	242	495	12
**KOG**	1	85	218	40
**KOMNZO**	3	63	94	118
**MJK**	57	539	958	98
**MON**	52	534	823	1037
**MUX**	23	40	107	136
**MWF**	9	55	68	26
**ROP**	6	106	163	7
**SJO**	7	77	107	1
**VRA**	5	48	82	232
**YUZ**	52	632	1145	210

SI-2.5 Societal: Society size and esotericity

We started out with the aim to include society size as an additional predictor variable. It has been argued that in smaller societies, people may know all other community members ([21], inter alia) and therefore may potentially have more comprehensive knowledge of kinship relationships in their own community. Thus, they may show a greater propensity for the use of kinship terms in reference. For this purpose and as shown in Table S6, we classified our languages as falling into society size bands based on the logarithmic scale standardly applied to population size (cf., e.g., [22]) and categorised them as belonging to either small societies (1,000 or fewer people) or large societies (more than 1,000 people). We did not further subdivide the group of large societies, as there is no evidence in the literature that indicates that progressively larger societies have less connection to their kin or social networks: you are just as unlikely to know everyone (and their family relationships) in a society of one million as in a society of ten million people. Hence, we opted for a binary classification that is not only motivated but also constitutes the strongest test case for the society size predictor, as a statistical effect is more likely.

As a proxy for society size we followed standard practice (cf. [23]), with population of a cultural group defined by their identification with the language used. This number better reflects society size than strict language user counts, as our data includes several multilingual populations and endangered languages, where only a portion of the society actually use the language, and languages where language user counts have changed dramatically over the past decade. Five of our languages (23%) were classified as being part of small societies (Dalabon, Komnzo, Matukar Panau, Sanzhi Dargwa, Vera'a) and the rest as large (see Table S6, showing the estimated society size bands, their assigned class and the languages in the study that belong to each band).

Table S6. Society size estimates (small society data rendered in italics)

Society size band	Class	Languages
1,000 and less	*small*	*Dalabon, Komnzo, Matukar Panau, Sanzhi Dargwa, Vera'a*
1,000-10,000	large	Auslan, Idi, Murrinhpatha, Roper Kriol, Yurakaré
10,000-100,000	large	Avatime, Duna, Kogi, Ku Waru, Sibe
100,000-1,000,000	large	Jinghpaw
1,000,000-10,000,000	large	Balinese, Khalkha-Mongolian, Ilokano
10,000,000 and above	large	English, German, Japanese

There are thus substantially fewer small societies in our study compared to large societies, which resulted in insufficient power in our statistical model to investigate this further. We find, however, that both small and large societies use high and low proportions of kinship terms, and that society size as operationalised here (with all its challenges) is unlikely to explain users' kinship term choices (see Fig S3). This result echoes the finding in [24] that society size may be less important than other social factors to the makeup of a kinship lexicon.

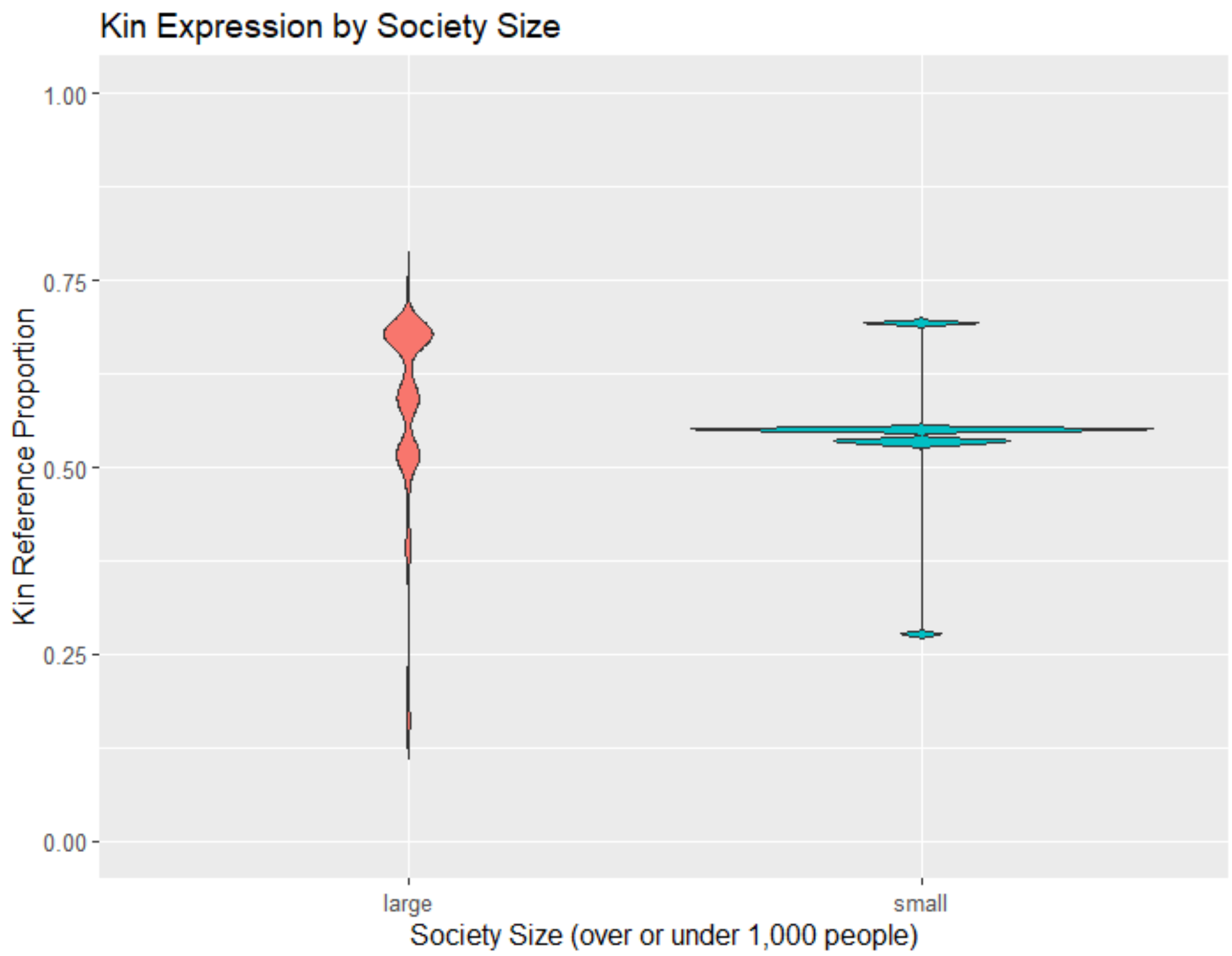

Fig S3. Kin reference proportion, separated by society size

Following this, we tried another approach to capturing the idea that knowing everyone in one's community might make kinship term usage more likely due to comprehensive knowledge of people's kinship relationships. This time, we categorised the languages into esoteric/inward ('users know everyone and are thus likely to know kin relationships') and exoteric/outward ('users do not know everyone and are thus likely to not know kin relationships') (cf. [25, 26]). Such a classification is different from society size, as even users within a nominally large society may typically only operate in a small community context. That is, a language classified as large might nevertheless be best classified as inward. As before, we opted for a binary classification (see Table S7), even though, as [26] states, esotericity forms a continuum.

Table S7. Esotericity classification

ISO	Esotericity
**ASF**	inward
**AVN**	inward
**BAN**	outward
**DAL**	inward
**DAR**	inward
**DEU**	outward
**DUC**	inward

**ENG**	outward
**IDI**	inward
**ILO**	outward
**JPN**	outward
**KAC**	inward
**KOG**	inward
**KOMNZO**	inward
**MJK**	inward
**MON**	inward
**MUX**	inward
**MWF**	outward
**ROP**	outward
**SJO**	outward
**VRA**	inward
**YUZ**	inward

Even though the distribution appeared to be more promising, sufficient power in our statistical model was not reached and we hence could not include this variable either. Fig S4, however, presents the kin expression proportion, separated by esotericity categories. In contrast to what we expected, namely that speakers or signers of inward languages tend to use more kin expressions due to extensive kin relationship knowledge, we see that that the kin reference proportion is lower for inward than for outward languages.

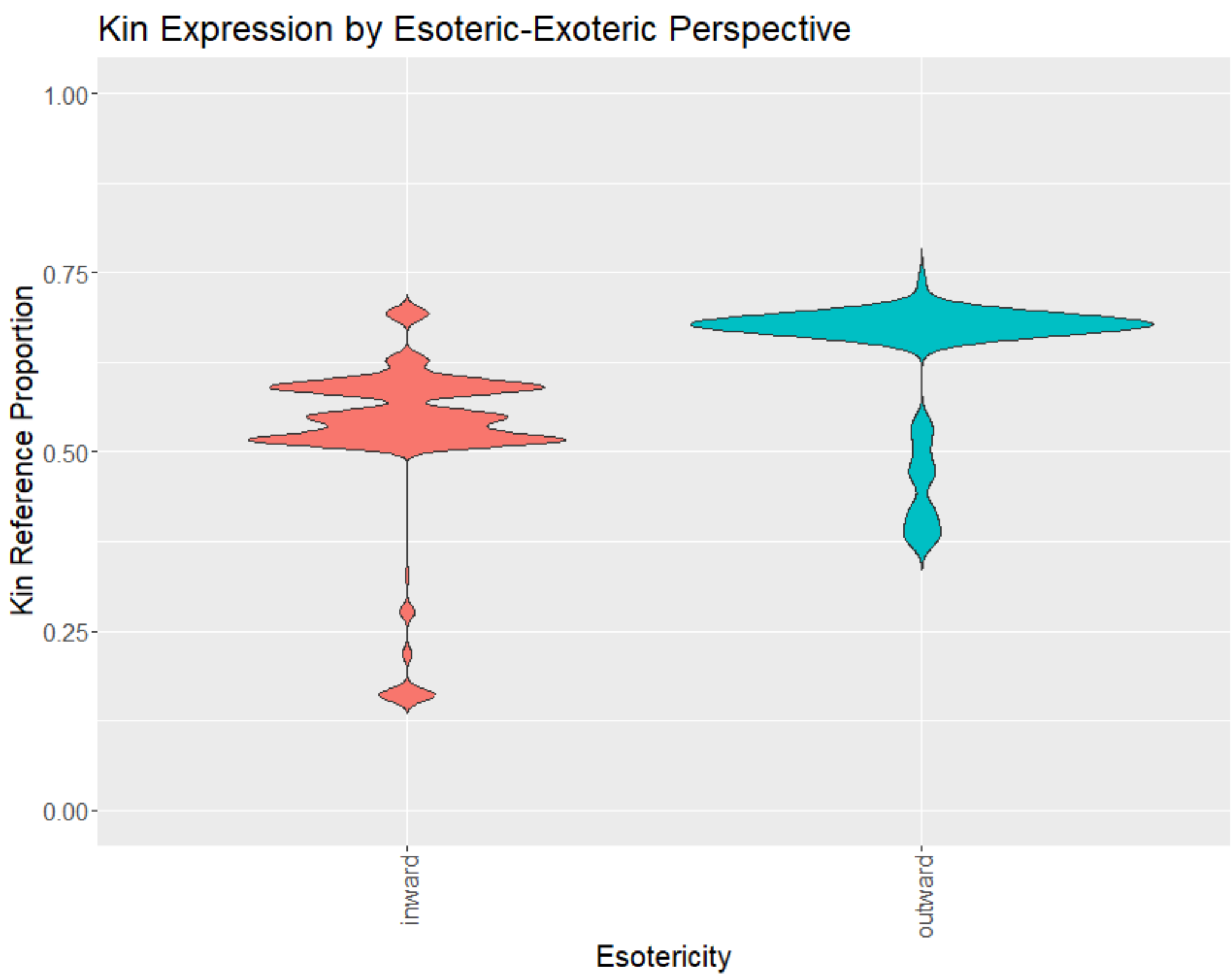

Fig S4. Kin reference proportion, separated by esotericity

SI-2.6: Language-distributional: Clade and macro-region

We also originally considered to include clade as a random effect and macro-region as a predictor, but both had too few instances (see Table 1 in the paper for our categorisation) to allow us to probe language family differences or regional differences. However, we find that there is a roughly even distribution of kinship terms uses versus other kinds of human referent uses across regions and language family clades, as shown in Figs S5 and S6.

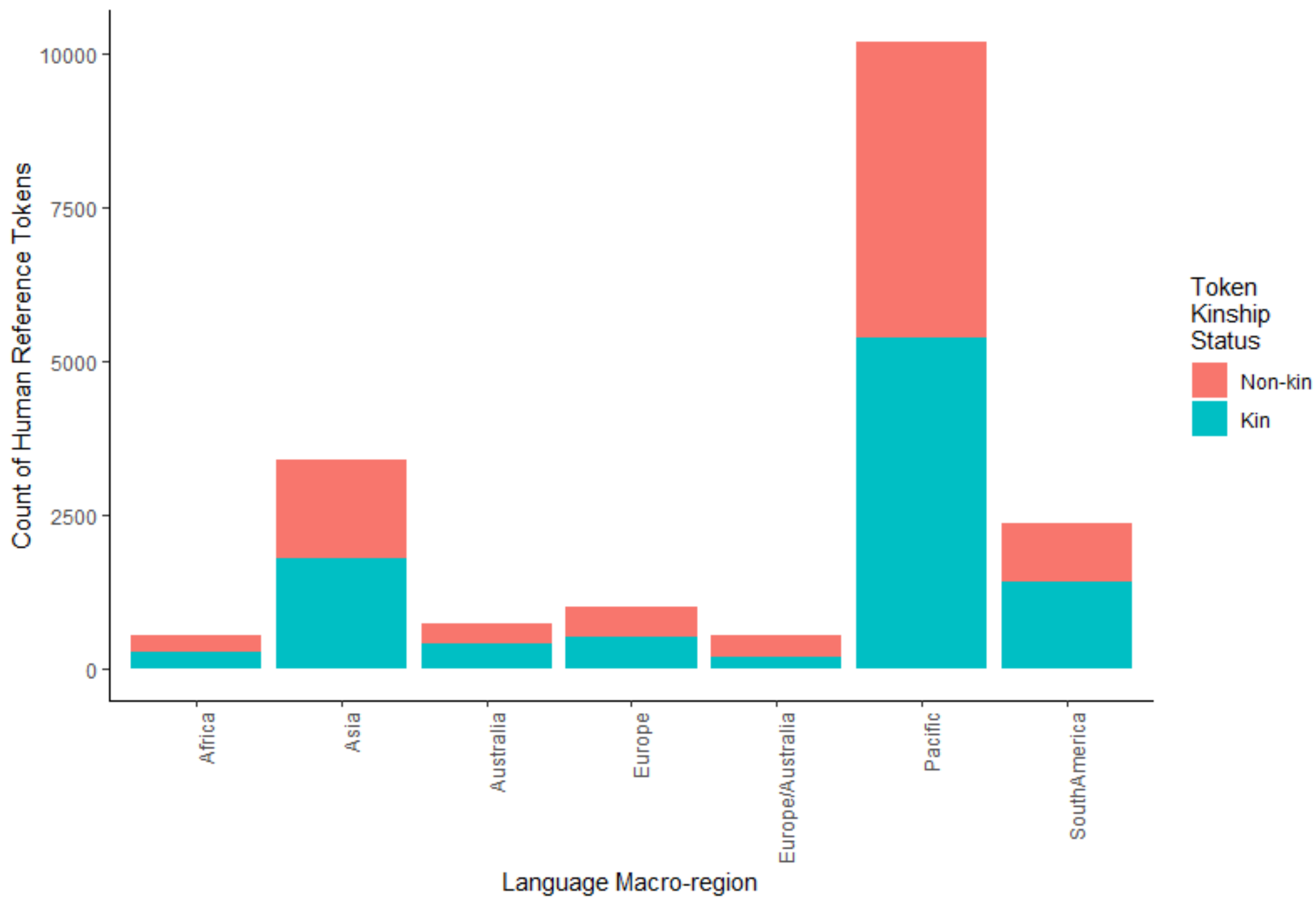

Fig S5. Kin vs non-kin reference tokens, by macro-region

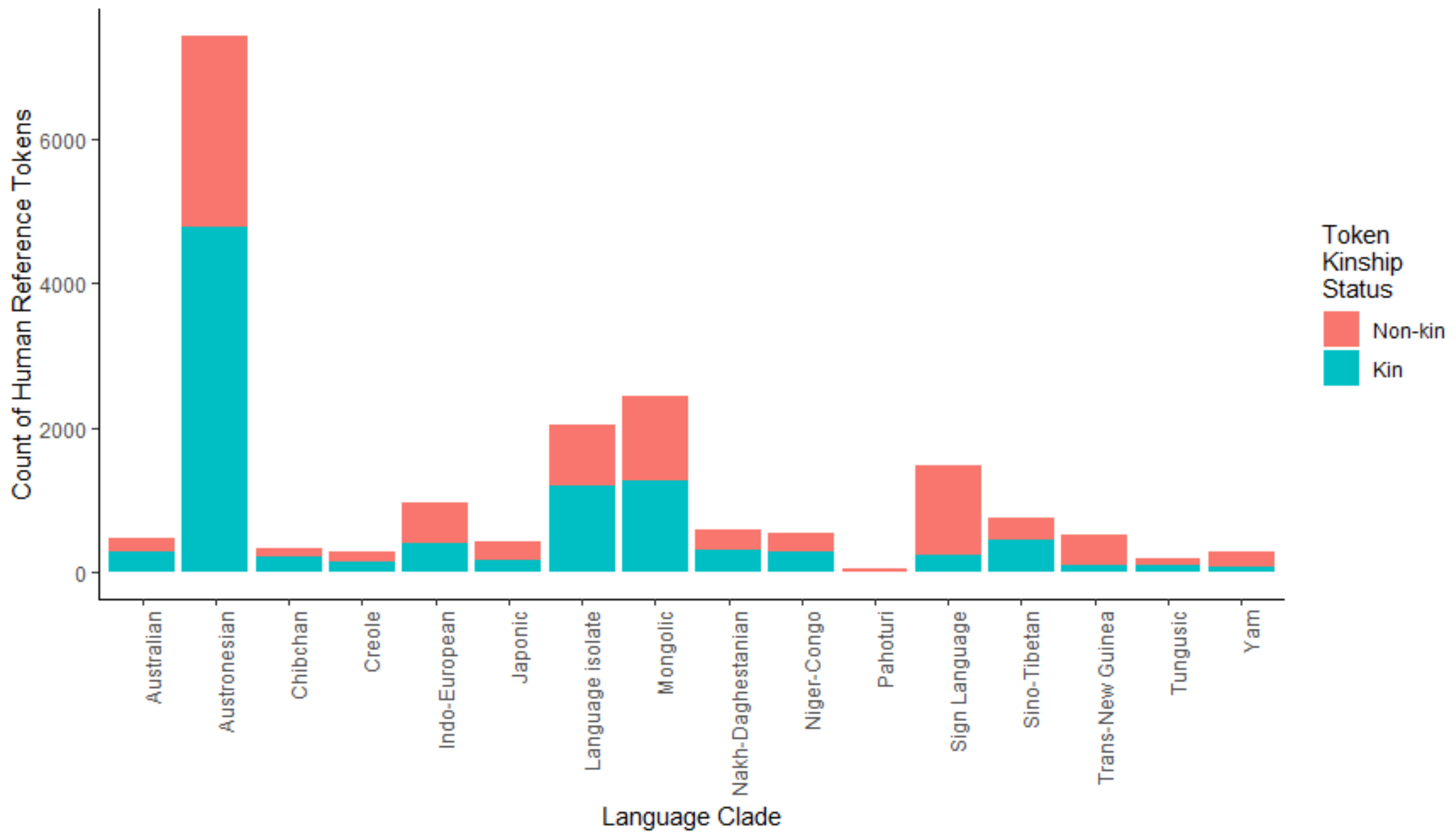

Fig S6. Kin vs non-kin reference tokens, by language clade

SI-2.7 Dependent variable – coding details

When annotating spoken language data, our researchers used the audible speech stream and not gestures or pointing that accompanies speech. When annotating signed language data, our researcher used the visual-gestural modality, which included lexified manual signs (woman, policeman), plurifunctional pointing signs (them-there, she-here), manual depictions and embodied enactments. Pointing signs, manual depictions and embodied enactments were included as potential instances of human reference for Auslan, but not for the spoken languages. We included words for friends as a kind of kinship term in our classification, since they too are relational terms for close human relationships. Pronominal expressions (*he, them, this one*) and address terms (*Mom!, Hey Bro!*) were excluded. Some instances were ambiguous between kin and generic categories. For example, the German word *Frau* means both ‘woman’ (a non-kin meaning) and ‘wife’ (a kin meaning), typically though not necessarily when combined with a possessive pronoun like *seine* ‘his’, but also attested unpossessed in our corpus, e.g. *und freudig empfangen würde von Frau und Kind* ‘and would be joyfully welcomed by wife and child’. Thus, unlike in languages where possessive marking is needed to evoke a kinship reading, in German the ‘wife’ meaning does not demand a possessive, cf. also *er kann eine Frau nicht ernähren* [he can a wife not support] ‘he cannot support a wife’ ([27], p. 96). In most cases, it was possible to infer the intended meaning from the context. Examples where it was not possible to determine from context if the meaning was generic or kinship were excluded from further analysis.

SI-3. Model building details

We constructed a multilevel mixed-effects logistic regression model to test predictors' effect on the frequency of kinship term usage. We included a random intercept for language, so that an average use of kinship terms was estimated for each language (*n* = 22). Nested within language are the session groups, i.e. groups of participants that together participate in a session (*n* = 100), as different groups varied somewhat in their frequency of kinship usage, even within a single language. Nested within the groups are the individual participants (*n* = 210). Participants had to produce four or more tokens to be included in the dataset. We also include stimuli cards (*n* = 17; 16 picture cards plus 'General/Non-specific') as a random effect to assess the effect of seeing particular scenes on people's kinship production (cf. Table S1 and Fig S7). Figs S7, S8 and S9 show the random intercepts for our multilevel model. Coefficients in our model (Table S8) show deviation away from each intercept. Table S9 provides the correlation of fixed effects and Table S10 the model fit. The model specification in R syntax is: kinL_full<-glmer(kin~ (1|CARD_1)+ (1|ISO/Group/Individuals) + kintax.score+scale(sib.terms)+Configuration2+discourse ,family=binomial,data=soccog.kn3,control = glmerControl(optimizer ="bobyqa")).

Table S8. Results of mixed-effects generalized linear regression

	b	*SE*	*Z*	*p*
Intercept	-1.75	0.42	-4.17	< 0.0001
Kintax score	0.41	0.12	3.56	< 0.001
Sibling term complexity	0.18	0.12	1.48	0.139
Configuration = peer	0.28	0.43	0.66	0.509
Configuration = intergenerational	1.54	0.42	3.87	< 0.001
Configuration = general	0.96	0.63	1.50	0.127
Discourse type = narrative	0.54	0.04	13.89	< 0.0001

Note: Positive coefficients in the second column (*b*) show that a factor is associated with higher kinship expression, negative coefficients are associated with non-kinship terms and coefficients close to zero are neutral. The reference level (comparison) for Configuration is Solitary and for Discourse Type it is conversation. Data token number after exclusions is *n* = 18,841. *SE* indicates Standard Error, the approximate standard deviation of the estimate. *Z* indicates the *Z*-score, a measure of how many standard deviations away the estimate is from the mean. Random effect variance and standard deviation are: Language variance (*n* = 22): 0.16 ± 0.40, Group: Language variance (*n* = 100): 0.23 ± 0.48, Individual: Group: Language variance (*n* = 210): 0.17 ± 0.41, Stimuli Card (*n* = 17): 0.27 ± 0.52.

Table S9. Correlation of fixed effects

	Intercept	Kintax score	Sibling term complexity	Configuration = general	Configuration = intergenerational	Configu-ration = peer
Kintax score	-0.35					
Sibling term complexity	0.16	-0.31				

Configuration = general	-0.52	0.00	0.00			
Configuration = intergenerational	-0.81	0.00	0.00	0.52		
Configuration = peer	-0.78	0.00	0.00	0.50	0.78	
Discourse type = narrative	-0.03	0.02	-0.02	-0.02	0.00	0.00

Table S10. Model fit

AIC	BIC	logLik	deviance	df.resid
21870.7	21957	-10924.4	21848.7	18830

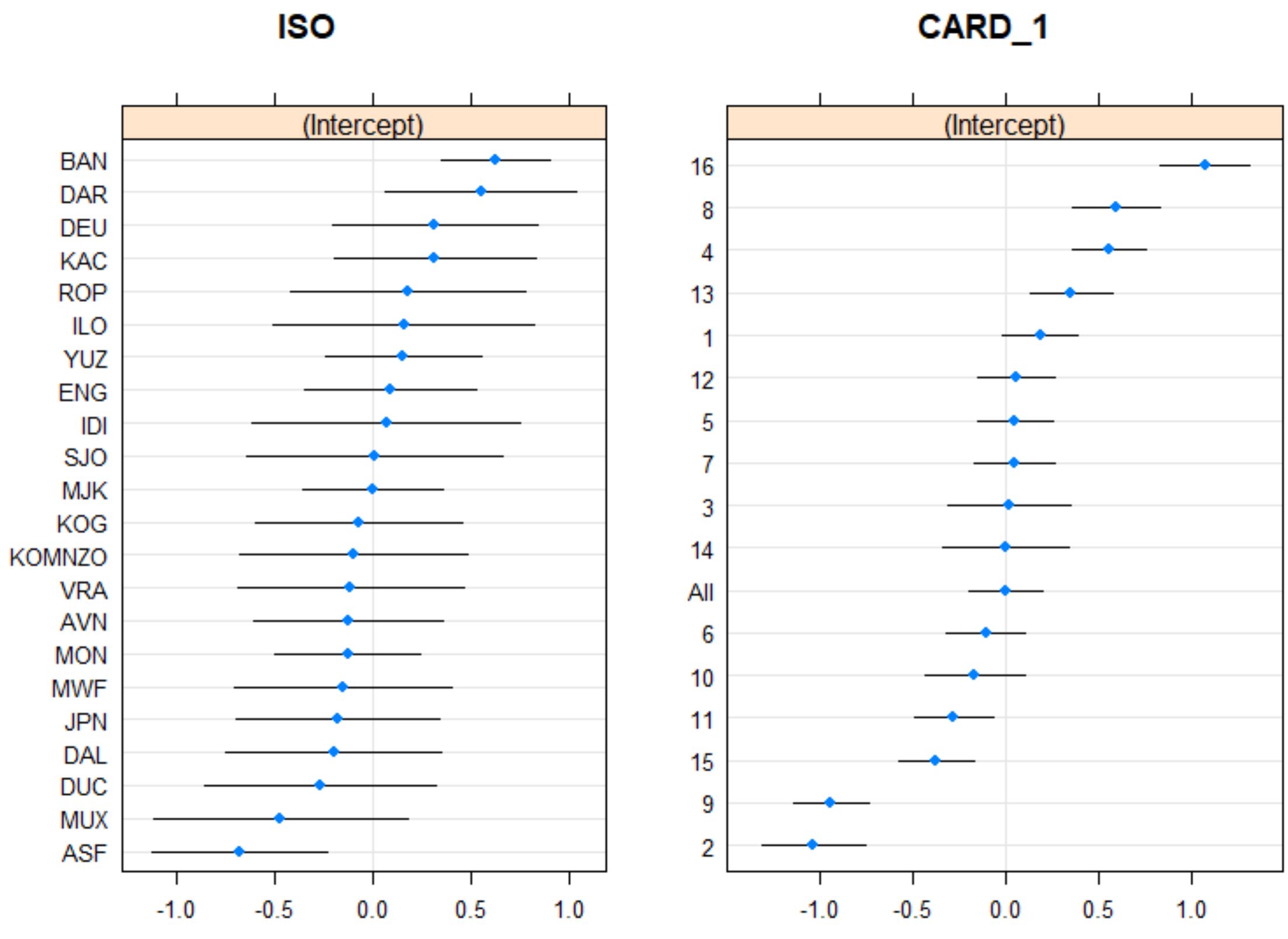

Fig S7. Model random intercepts for language (ISO) and stimuli picture (CARD)

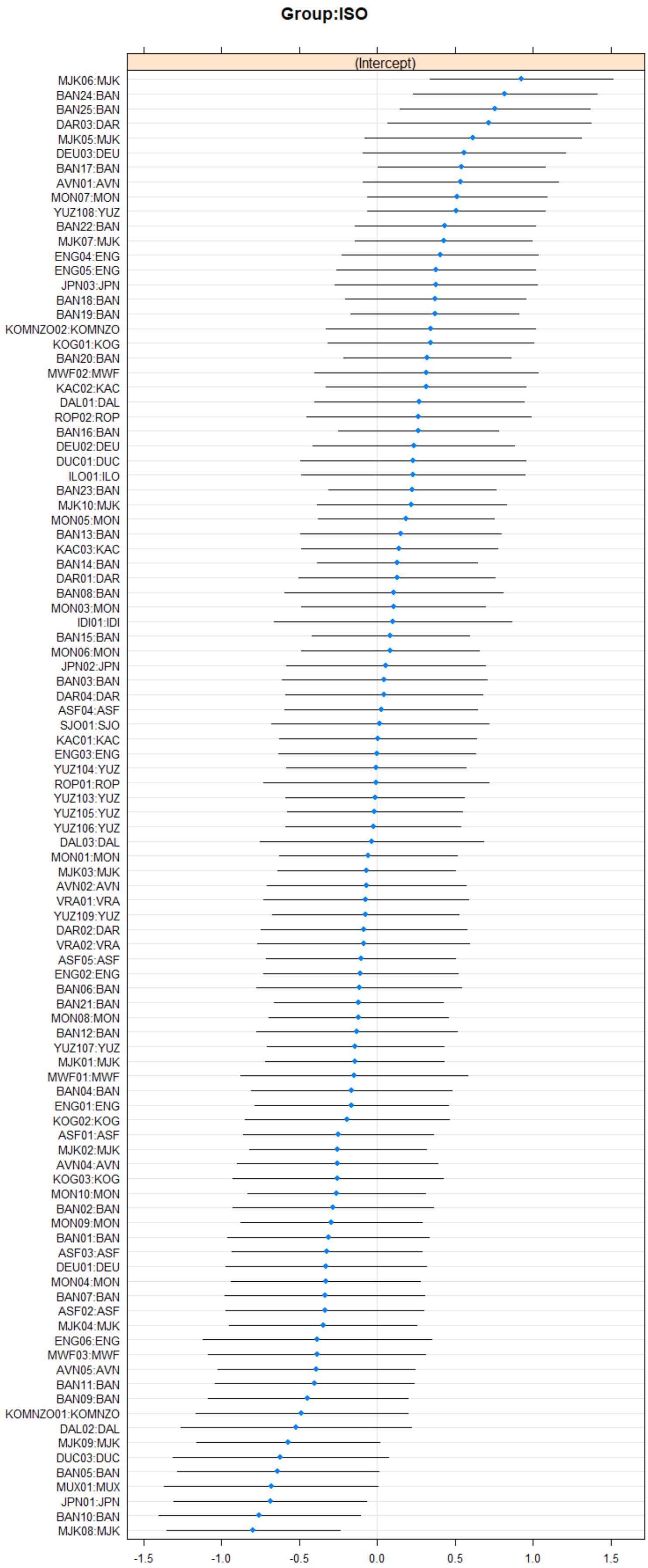

Fig S8. Model Random Intercepts for Session Group

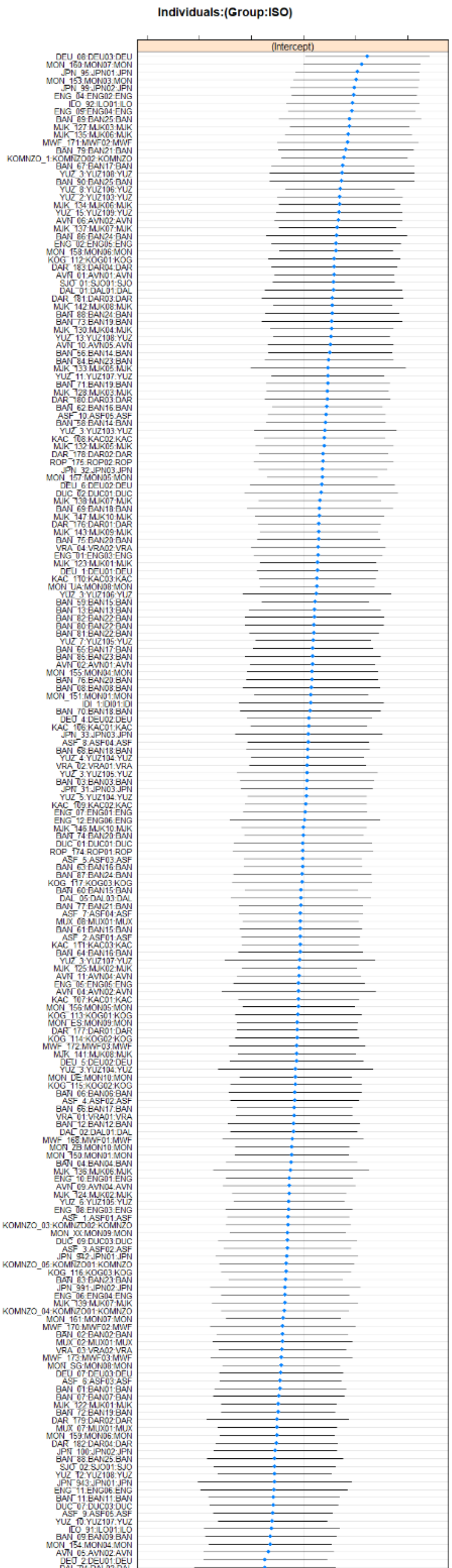

Fig S9. Model Random Intercepts for Individual Participant

SI-4. Stimulus Materials

The stimulus materials for the Family Problems Picture Task (described in more detail in [28]) consist of a set of 16 original picture cards drawn in black and white, all of which are shown in Fig S10 in their presentation order. The pictures can be interpreted as depicting different kin and non-kin configurations (see SI-2.4), and resonate with a wide range of language communities due to a 'generic' village environment and depictions of familiar societal and governmental structures. While illustrating a story, the cards are presented to participants in the modified order shown in Fig S10 that encourages negotiation between task participants about the characters and their social relations in the conversation phases of the task (description and arrangement). In the following narration phases, participants build their narrations on these jointly negotiated relations, introducing them as a given to the third (naïve) participant.

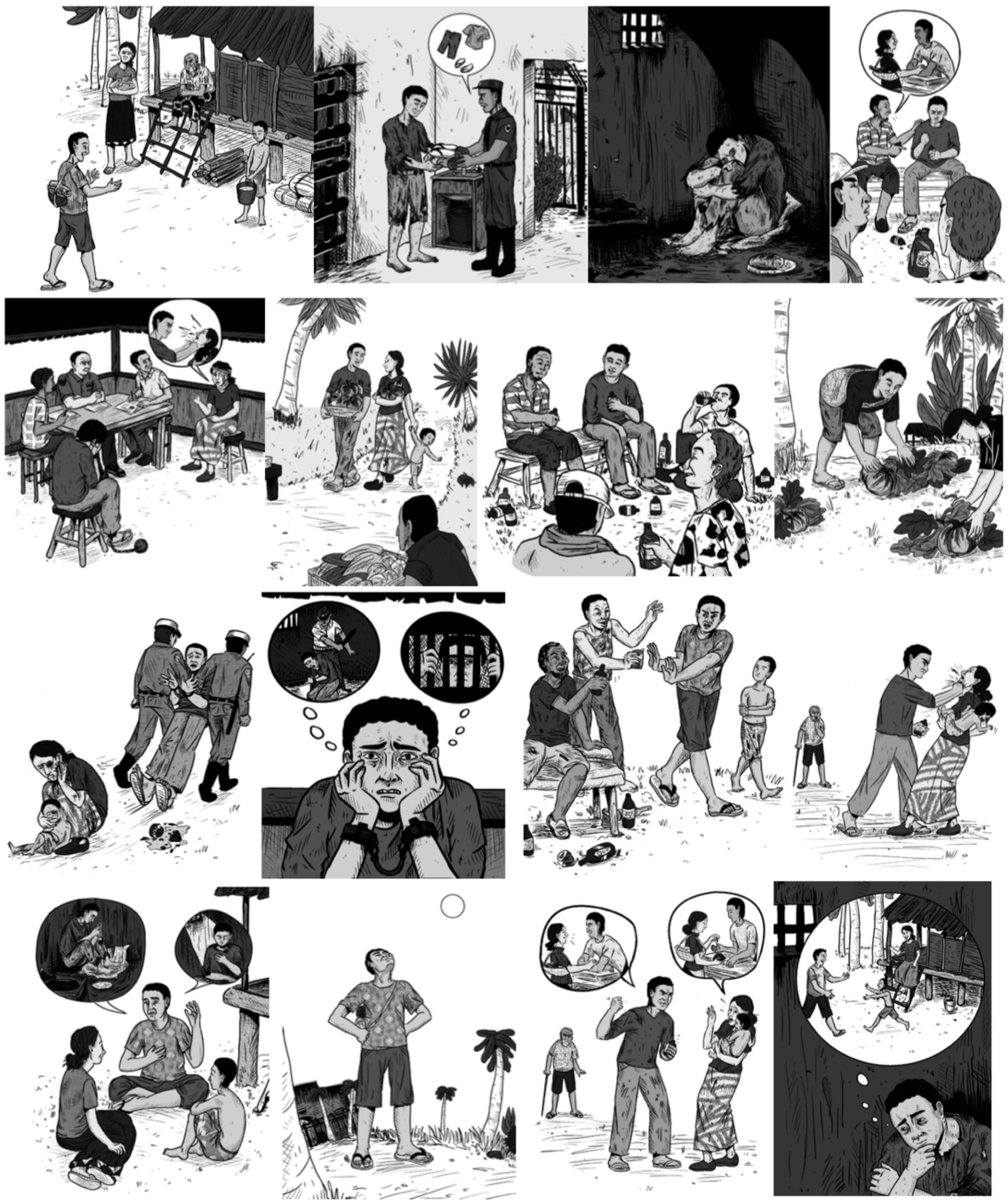

Fig. S10. Stimuli from the Family Problems Picture Task (presentation order) (The figures are in the public domain and available at https://scopicproject.wordpress.com/run-the-task/ and https://scopicproject.wordpress.com/wp-content/uploads/2018/01/stimuli-pictures.pdf.)

SI-5. SI References

1. Kockelman P. Inalienable possession as grammatical category and discourse pattern. Stud Lang. 2009;33(1): 25–68.
2. Dahl Ö, Koptjevskaja-Tamm M. Kinship in grammar. In: Baron I, Herslund M, Sørensen F, editors. Dimensions of possession. Amsterdam/Philadelphia: John Benjamins; 2001. pp. 201–226. https://doi.org/10.1075/tsl.47.12dah
3. Evans, N. Social cognition in Dalabon. In: Barth D, Evans N, editors. Social Cognition Parallax Interview Corpus (SCOPIC) (Language Documentation & Conservation Special Publication No. 12). Honolulu: University of Hawai'i Press; 2021. pp. 22-84. http://hdl.handle.net/10125/24743
4. Guntsetseg, D. Differential case marking in Mongolian. Wiesbaden: Harrassowitz; 2016.
5. Clynes, A. 1995. Topics in the phonology and morphosyntax of Balinese. PhD dissertation, Australian National University;1995. https://doi.org/10.25911/5d77865d38e15.
6. Okamoto S. Social context, linguistic ideology, and indexical expressions in Japanese. J Pragmat. 1997;28(6): 795–817.
7. Shibatani M. The Languages of Japan. Cambridge: Cambridge University Press; 1990.
8. Blythe J. Preference organization driving structuration: Evidence from Australian Aboriginal interaction for pragmatically motivated grammaticalization. Language. 2013;89(4): 883–919.
9. Street CS. An Introduction to the Language and Culture of the Murrinh-Patha. Darwin: Summer Institute of Linguistics; 1987.
10. Alpher B. Dalabon dual-subject prefixes, kinship categories, and generation-skewing. In: Heath J, Merlan F, Rumsey A, editors. Languages of kinship in Aboriginal Australia, Sydney: The University of Sydney; 1982. pp. 19–30.
11. Evans N. Dyad constructions. In: Brown K, editor. Encyclopaedia of Language and Linguistics, Vol. 4. Amsterdam: Elsevier; 2006. pp. 24–27.
12. Koch H. Kinship categories in Kaytej pronouns. In: Heath J, Merlan F, Rumsey A, editors. Languages of kinship in Aboriginal Australia, Sydney: The University of Sydney; 1982. pp. 64–71.
13. Merlan F, Heath J. Dyadic kinship terms. In: Heath J, Merlan F, Rumsey A, editors. Languages of kinship in Aboriginal Australia, Sydney: The University of Sydney; 1982. pp. 107–124.

14. Kemp C, Xu Y, Regier T. Semantic typology and efficient communication. Annu Rev Linguist. 2018;4: 109–128.
15. Boas F. Introduction. In: Boas F, editor. Handbook of American Indian Languages, Vol. 1. Washington: Government Printing Office; 1911. pp. 1–83.
16. von der Gabelentz G. Die Sprachwissenschaft, ihre Aufgaben, Methoden und bisherigen Ergebnisse: Zweite, vermehrte und verbesserte Auflage. Graf von der Schulenburg A, editor. Leipzig: Tauchnitz; 1901.
17. Murdock GP. Social Structure. New York: Macmillan; 1949.
18. Kemp C, Regier T. Kinship categories across languages reflect general communicative principles. Science. 2012;336: 1049–1054.
19. Nerlove S, Romney AK. Sibling terminology and cross-sex behavior. Am Anthropol. 1967;69(2): 179–187.
20. Hirtzel V. Le maître à deux têtes: Une ethnographie du rapport à soi yuracaré (Amazonie bolivienne). PhD dissertatin, École des Hautes Études en Sciences Sociales Paris; 2010. Available from: https://theses.hal.science/tel-01984217
21. Dunbar RI. The social brain: Psychological underpinnings and implications for the structure of organizations. Curr Dir Psychol Sci. 2014;23(2): 109–114.
22. Wichmann S, Holman EW. Population size and rates of language change. Hum Biol. 2009;81(2/3): 259–274. http://www.jstor.org/stable/41466601
23. Kirby KR, Gray RD, Greenhill SJ, Jordan FM, Gomes-Ng S, Bibiko H-J, Blasi DE, Botero CA, Bowern C, Ember CR, Leehr D, Low BS, McCarter J, Divale W, Gavin MC. D-PLACE: A global database of cultural, linguistic and environmental diversity. PLoS ONE. 2016;11(7): e0158391. https://doi.org/10.1371/journal.pone.0158391
24. Rácz P, Passmore S, Jordan FM. Social practice and shared history, not social scale, structure cross-cultural complexity in kinship systems. Top Cogn Sci. 2020;12: 744–765.
25. Wray A, Grace GW. The consequences of talking to strangers: Evolutionary corollaries of socio-cultural influences on linguistic form. Lingua. 2007;117(3): 543–578. https://doi.org/10.1016/j.lingua.2005.05.005
26. Lupyan G, Dale R. Language structure is partly determined by social structure. PLoS ONE. 2010;5(1): e8559. doi:10.1371/journal.pone.0008559
27. Strauss GL. A grammar of the German language, adapted for the use of English students, from Heyse's theoretical and practical German grammar, 2nd ed. London: Weale; 1856.
28. San Roque L, Gawne L, Hoenigman D, Miller JC, Rumsey A, Spronck S, Carroll A, Evans N. Getting the story straight: Language fieldwork using a narrative problem-solving task. Language Documentation & Conservation. 2012;6: 135–174.
